# Effects of elevated ozone and warming on terpenoid emissions and concentrations of Norway spruce depend on needle phenology and age

**DOI:** 10.1093/treephys/tpac019

**Published:** 2022-02-19

**Authors:** Minna Kivimäenpää, Johanna Riikonen, Hanna Valolahti, Häikiö Elina, Jarmo K Holopainen, Toini Holopainen

**Affiliations:** Department of Environmental and Biological Sciences, University of Eastern Finland, PO Box 1627, Kuopio 70211, Finland; Natural Resources Institute Finland, Suonenjoki 77600, Finland; Natural Resources Institute Finland, Suonenjoki 77600, Finland; Department of Environmental and Biological Sciences, University of Eastern Finland, PO Box 1627, Kuopio 70211, Finland; Ramboll, Niemenkatu 73, Lahti 15140, Finland; Department of Environmental and Biological Sciences, University of Eastern Finland, PO Box 1627, Kuopio 70211, Finland; South Savo Centre for Economic Development, Transport and the Environment, PO Box 164, Mikkeli 50101, Finland; Department of Environmental and Biological Sciences, University of Eastern Finland, PO Box 1627, Kuopio 70211, Finland; Department of Environmental and Biological Sciences, University of Eastern Finland, PO Box 1627, Kuopio 70211, Finland

**Keywords:** biogenic organic volatile compound (BVOCs), climate change, FACE, monoterpene, needle age, needles, Norway spruce, open-field exposure, ozone, photosynthesis, *Picea abies*, seasonal changes, sesquiterpene, warming

## Abstract

Norway spruce (*Picea abies* (L.) Karst) trees are affected by ongoing climate change, including warming and exposure to phytotoxic levels of ozone. Non-volatile terpenoids and volatile terpenoids (biogenic organic volatile compounds, BVOCs) protect spruce against biotic and abiotic stresses. BVOCs also affect the atmosphere’s oxidative capacity. Four-year-old Norway spruce were exposed to elevated ozone (EO) (1.4 × ambient) and warming (1.1 °C + ambient air) alone and in combination on an open-field exposure site in Central Finland. Net photosynthesis, needle terpenoid concentrations and BVOC emissions were measured four times during the experiment’s second growing season: after bud opening in May, during the mid-growing season in June, and after needle maturation in August and September. Warming increased terpene concentrations in May due to advanced phenology and decreased them at the end of the growing season in matured current-year needles. Ozone enhanced these effects of warming on several compounds. Warming decreased concentrations of oxygenated sesquiterpenes in previous-year needles. Decreased emissions of oxygenated monoterpenes by warming and ozone alone in May were less prominent when ozone and warming were combined. A similar interactive treatment response in isoprene, camphene, tricyclene and α-pinene was observed in August when the temperature and ozone concentration was high. The results suggest long-term warming may reduce the terpenoid-based defence capacity of young spruce, but the defence capacity can be increased during the most sensitive growth phase (after bud break), and when high temperatures or ozone concentrations co-occur. Reduced BVOC emissions from young spruce may decrease the atmosphere’s oxidative capacity in the warmer future, but the effect of EO may be marginal because less reactive minor compounds are affected.

## Introduction

Norway spruce (*Picea abies* (L.) Karst.) is an ecologically and economically important, widely distributed coniferous tree species in North and Central Europe (Caudullo et al. 2016). Norway spruce is affected by ongoing climate change, including warming and elevated concentrations of tropospheric ozone, which is an air pollutant and a greenhouse gas (Monks et al. 2015). Depending on future greenhouse gas emissions, it is predicted the air temperature will increase by 1.0–5.7 °C globally by the end of this century compared with 1850–1900, and the temperature increase will be higher in northern latitudes (Allan et al. 2021). Warming by 1–6 °C has been shown to advance the shoot ontogeny of Norway spruce, while both reductions and increments in photosynthesis and the growth of mature and young spruce have been reported in experiments conducted in the field (Hänninen et al. 2007, Hall et al. 2009, Riikonen et al. 2012, Kivimäenpää et al. 2017, Nissinen et al. 2020). Ozone concentrations increased from a level of 15–20  p.p.b. in the 1950s to one of 30–55 p.p.b. in the 2010s in Europe (Cooper et al. 2014). It has been shown that prevailing or moderately elevated ozone (EO) concentrations cause several negative effects on young and mature Norway spruce, including cellular alterations and visible symptoms on needles or reductions in photosynthesis and growth (Wallin et al. 2002, Wieser et al. 2002, Kivimäenpää et al. 2005, Karlsson et al. 2006). Despite the slightly decreased ozone concentrations in Europe since 2000, the stomatal uptake of ozone has increased, indicating that ozone remains a threat to vegetation (Proietti et al. 2020).

Terpenoids are plant secondary compounds that are stored in the resin duct cavity and synthesized in the epithelial cells of the resin ducts in Norway spruce needles. Volatile terpenoids, monoterpenes (MTs) (C10) and sesquiterpenes (STs) (C15) are emitted from the storage structures (pool emissions) and counted as biogenic volatile organic compounds (BVOCs). Isoprene (C5) emissions and a substantial proportion of spruce MT emissions originate from de novo synthesis in the mesophyll tissue, depending on light availability and photosynthesis (Ghirardo et al. 2010). BVOCs from intact needles are released via the stomata and can be affected by stomatal conductance, as well as diffusion through the cuticle, while damage (such as herbivory) causes a burst of emissions mainly from the resin ducts (Loreto and Schnitzler 2010).

Terpenoids, both in tissue and in volatile blends, are essential in conifer defence against biotic stresses by affecting resin fluidity, having direct repellent or toxic effects, affecting the orientation of herbivores or their natural enemies, or acting as signal molecules activating defence against pathogens (Trapp and Croteau 2001, Mumm and Hilker 2006, Lundborg et al. 2016, Celedon and Bohlmann 2019, Frank et al. 2021). Terpenoids also offer protection against oxidative stress such as ozone and heat, possibly by scavenging oxidants or reactive oxygen species (ROS) in the intercellular space or boundary layer, maintaining membrane functionality or acting as signal molecules for the inducing synthesis of other defence compounds (Sharkey 1996, Velikova et al. 2005, Loreto and Schnitzler 2010, Monson et al. 2021). BVOCs react with oxidants, including ozone, in the atmosphere, forming secondary organic aerosols (SOA) (Mentel et al. 2013, Joutsensaari et al. 2015) which make light more diffuse and increase cloud condensation nuclei, promoting cloudiness (Paasonen et al. 2013, Ehn et al. 2014). It is predicted these processes will have a cooling effect on the climate in the boreal region in future climatic conditions (Spracklen et al. 2008, Paasonen et al. 2013).

Climate change can affect both terpene concentrations of conifer needles and their emissions (Holopainen et al. 2018) and thus the defence capability against stresses, as well as climate feedback via SOA formation. The results of short-term experiments, laboratory experiments, or experiments using a high ozone concentration or temperature may not be feasible to predict climate change effects on the terpene chemistry of boreal conifers, because the results contrast with those from long-term field experiments with modest temperature and ozone elevations (Table 1).

**Table 1:** Responses of Norway spruce needle MT concentrations and MT emission rates to EO and ET.

Factor	Experimental conditions	Response	References
Ozone	Growth chamber, 300–600 p.p.b. (2–3 days), 150 p.p.b. (22 days)	Concentration unaffected	Kainulainen et al. 1995
Ozone	Open-field, 1.2–1.7 × ambient (3 growing seasons), monthly 7-h means up to 57 p.p.b. (extrapolated), AOT40 up to 25 p.p.m. h_−1_	22–45% increase in individual compound concentrations, end of last growing season	Kainulainen et al. 2000
Ozone	Greenhouse + branch chamber, 200 p.p.b. (ca 1 h)	Emissions unaffected	Esposito et al. 2016
Ozone	Growth chamber, 80 p.p.b. (5–6 weeks, spring and summer)	78% increase in nMT, 158% increase in oMT emissions in summer	Yu and Blande 2021
Ozone	Open-field, 1.4 × ambient (1 growing season), 36 p.p.b., max. Hourly concentration 62 p.p.b.	2-fold increase in emissions, mid-growing season	Kivimäenpää et al. 2013
Temperature	Growth chamber, +4 °C (50 days)	67% increase in concentration	Sallas et al. 2003
Temperature	Laboratory, temperature ramp from 23 to 35 °C (hours)	ca 10% increase in emissions	Filella et al. 2007
Temperature	Greenhouse + branch chamber, 30 vs 40 °C (ca 1 h)	170% increase in emissions	Esposito et al. 2016
Temperature	Open-field, +1.3 °C (1 growing season)	Transient increase in oMTs (compound profile change), mid-growing season	Kivimäenpää et al. 2013

Abbreviations: AOT40: Accumulated exposure over threshold 40 p.p.b. (Mills et al. 2017), nMT = non-oxygenated MT, oMT = oxygenated MT.

Terpenoid concentrations and terpenoid emissions of Norway spruce show seasonal trends that can affect defence capacity and atmospheric oxidative reactivity during the growing season. Monoterpene concentrations are low in developing shoots but increase rapidly during needle maturation and decrease in the autumn (Schönwitz et al. 1990), but data on seasonal changes in ST concentrations are scarce. Bourtsoukidis et al. (2014) showed a decreasing trend of MT and ST emission rates from the spring to the autumn measured from a branch of a mature Norway spruce, whereas Wang et al. (2017) reported the highest emission in August and the lowest in September at the end of the growing season. It is unknown if long-term exposure to ozone and warming alone or in combination affects the seasonal changes in terpenoid chemistry, although previously observed changes in phenology, physiology and needle structure affecting terpenoid chemistry may suggest this. For example, earlier bud burst because of warming (Riikonen et al. 2012) could shift the seasonal BVOC emission peak in the spring (Bourtsoukidis et al. 2014). In addition, decreased photosynthesis due to ozone (Wallin et al. 1990) or warming (Kivimäenpää et al. 2013) in Norway spruce seedlings could reduce the photosynthesis-dependent synthesis of terpenoids and their de novo emissions (Ghirardo et al. 2010), especially at the end of the season because of the accumulating effect of both factors. Moreover, the reduction of resin canal size by warming (Kivimäenpää et al. 2017) can affect the size of resin and terpene storage and affect BVOC pool emissions from mature needles.

This study sought to reveal if moderately EO and warming or elevated temperature (ET) alone and in combination affect terpenoid concentrations and terpenoid emissions at the various phenological stages of Norway spruce during the growing season. The role of needle gas exchange linked with terpenoid synthesis and BVOC emissions (Ghirardo et al. 2010, Loreto and Schnitzler 2010) was also studied. BVOC emissions from the mid-growing season and needle gas exchange from the first year of the study have previously been published (Riikonen et al. 2012, Kivimäenpää et al. 2013). In this paper, acclimation to warming and EO was longer, and their combined effects on terpenoid concentrations were studied for the first time.

## Materials and methods

### Experimental setup

Norway spruce seedlings were exposed to EO concentrations and an elevated temperature (ET) alone and in combination in an open-field exposure site between 9 June 2009 and 15 September 2010 in Kuopio, Central Finland (62°53′42″ N, 27°37′ 30″ E, 80 m above sea level). The seedlings were 4 years old in 2010 when the measurements for this study were taken. The experimental setup was of a split-plot design and has been described in Riikonen et al. (2012) and Kivimäenpää et al. (2013, 2017). In brief, there were four circular (diameter 10 m) ambient ozone (AO) plots and four EO plots, and within each plot, there were two rectangular subplots (190 × 140 cm), one for ambient temperature (AT) and one for ET. In the subplots, seedlings grew in a soil bed containing a mixture of mull and sand (2:1) and were fertilized with 4 kg m^−3^ of Peatcare Slow Release 1 (Yara, Siilinjärvi, Finland) to give a total amount of 42.6 kg N ha^−1^ year^−1^. Each subplot had 24 experimental seedlings and 22 additional seedlings at the edges of the subplot to give similar conditions for root competition and shading at the beginning of the experiment. The seedlings were harvested for various measurements during the experiment (Riikonen et al. 2012, Kivimäenpää et al. 2013), keeping the light availability of the seedlings similar during the experiment.

**Figure 1.**
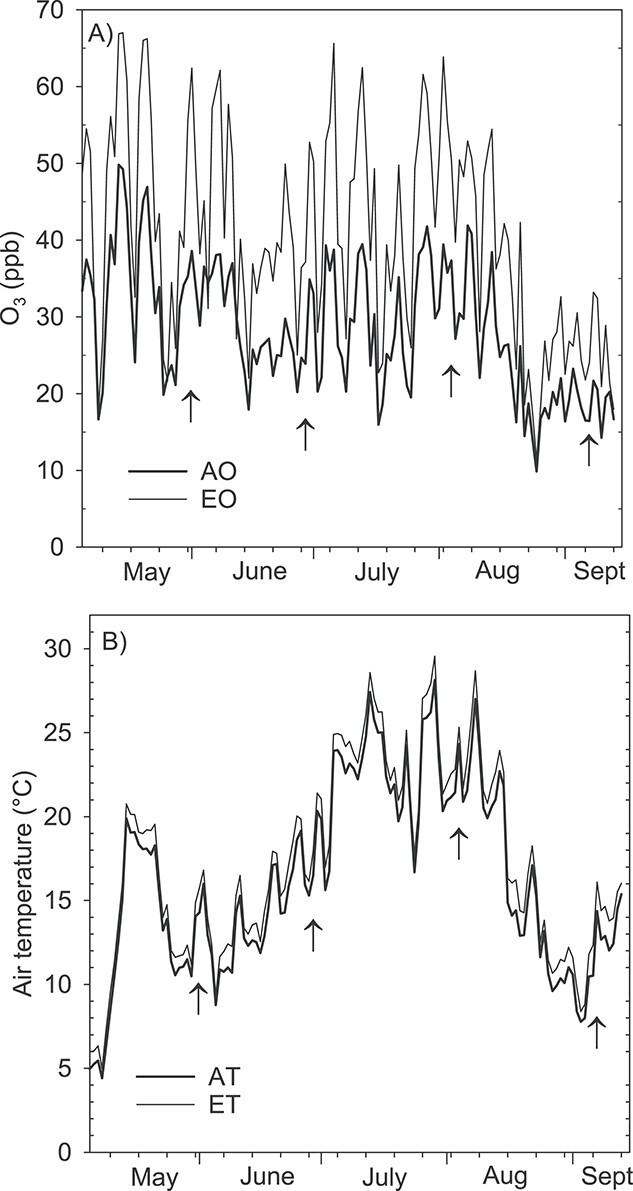
(A) Average ozone concentration as daytime average (8:00–22:00 h) from AO and EO plots (*n* = 4) and (B) average air temperatures as daily averages (0:00–24:00 h) from AT and ET plots (*n* = 8) in 2010 growing season. Arrows indicate dates when BVOCs from Norway spruce seedlings were collected. Shoot gas exchange was measured, and samples for terpene needle concentration analysis were collected 1 day later.

Ozone was released through vertical perforated tubes around the circular plots between 8:00 and 22:00 h (Eastern European Summer Time, UTC + 3) every day during the 2009 and 2010 growing seasons, except during rain, very low wind velocity (<0.1 m s^−1^), or if the AO concentration was below 10 p.p.b. The treatments resulted in 1.4 × mean AO concentrations in the elevated plots in both years, and the cumulative ozone exposure index AOT40 (Accumulated Exposure Over Threshold 40 p.p.b.) (Mills et al. 2017) was 0.14 p.p.m.h AO and 4.71 p.p.m.h EO in 2009 and 1.39 p.p.m.h AO and 13.48 p.p.m.h EO in 2010 (Kivimäenpää et al. 2017). Figure 1A shows the daytime average ozone concentration during the 2010 growing season.

The ET treatment was conducted with infrared (IR) heaters and continued throughout the study period, including in the winter (Riikonen et al. 2012). The air temperature elevation was 1.3 °C during the 2009 growing season and 1.1 °C in 2010. Infrared heaters warm materials more than air and it was estimated the needle surface temperature increased by an additional 1 °C under ET (Kivimäenpää et al. 2013). The cumulative temperature sums (degree days) were 1345 (AT) and 1467 (ET) in 2009 and 1497 (AT) and 1585 (ET) in 2010 (Kivimäenpää et al. 2017). The daily average temperatures during the 2010 growing season are shown in Figure 1B.

The topsoil’s water content was measured daily with a soil moisture sensor (Theta probe, type ML2, Delta-T Devices, Cambridge, UK), and seedlings were kept well-watered throughout the growing seasons (Kivimäenpää et al. 2017). Winter warming increased the proportion of seedlings with visible needle damage in the spring of 2010 (Kivimäenpää et al. 2014), but green, healthy-looking seedlings were selected for the current study. The seedlings showed no visible resin droplets that could increase BVOC emissions (Eller et al. 2013). Neither ozone nor warming during the growing seasons caused visible symptoms in needles. The seedlings were not pesticide-treated. In August 2010, a few seedlings from warming treatments had a few aphids (probably *Cinara pilicornis*) in the stems of the current-year shoots. It was not always possible to select aphid-free seedlings for the measurements.

The following abbreviations are used for the treatments: AOAT = AO, AT; AOET = AO, ET; EOAT = EO, AT; EOET = EO, ET.

### Growth and phenological stage of the seedlings during sampling

The BVOC emissions rates of the seedlings, terpenoid needle concentrations and shoot gas exchange were determined four times during the experiment’s second growing season, in weeks 22, 26, 31 and 36 in 2010. The BVOC emission was measured first, and the exact sampling dates were 31 May (week 22), 28 June (week 26), 2–3 August (week 31) and 6 September (week 36). Gas exchange measurements and sampling for terpenoid needle concentrations from the same seedlings were taken the next day. One or two seedlings were sampled from each subplot. However, only three subplots were sampled from the AOET and EOET treatments in weeks 22 and 26 and the AOET treatment in week 36. In total, 28–32 seedlings were measured each time. The seedlings were harvested after each sampling, and different seedlings were thus sampled on each sampling occasion. The length of the main shoot was measured, and Lammas growth was monitored at harvest (Figure S1 and Table S1 available as Supplementary data at *Tree Physiology* Online). In week 22, shoot and needle growth were ongoing, and the stems were fleshy and green. The length of the main shoot ranged from 0.6 to 9.7 cm. The main shoots were significantly longer in the ET than AT treatments in week 22 (Figure S1 available as Supplementary data at *Tree Physiology* Online). In the ET treatments, bud burst (Riikonen et al. 2012) took place 12 days before the week 22 sampling and in the AT treatments, 7 days. In week 26, the main shoot growth had ended, and buds were forming. In weeks 31 and 36, the needles had matured, and seedlings showed Lammas growth, which was increased by warming (Table S1 available as Supplementary data at *Tree Physiology* Online). The part of the seedling enclosed in a BVOC collection bag (see below) was cut and divided into current and older needles and stems and dried at +60 °C for 3 days, and dry weight (DW) was measured and used to calculate the BVOC emission rates. In week 22, due to the shoots’ early developmental stage, it was not always possible to detach the needles from the stems. A fresh, green stem was therefore included in needle DW in all samples in week 22. Table S1 available as Supplementary data at *Tree Physiology* Online shows the fractions of needles and stems in the BVOC collection bags in each treatment and on each sampling occasion. The collection bags for ET seedlings had a higher proportion of current-year growth and needles than AT seedlings in week 22 (Table S1 available as Supplementary data at *Tree Physiology* Online). A reduced shoot growth by ET was reflected as a lower proportion of current-year growth (especially needles) in the BVOC collection bags for ET seedlings in weeks 31 and 36 (Table S1 available as Supplementary data at *Tree Physiology* Online).

### BVOC emissions

The sampling methods and the analysis for determining BVOC emission rates were the same as described by Kivimäenpää et al. (2013). Briefly, the seedlings were enclosed inside pre-cleaned (+120 °C for 1 h) 2.5-l PET bags into which ozone-free, carbon-filtered replacement air (0.6 l min^−1^ during 15 min flushing, 0.3 l min^−1^ during 15 min sampling) was pumped with a battery-operated collection system. The 3-l air samples (0.2 l min^−1^ for 15 min) were pulled through an adsorbent-filled (Tenax TA 60/80) steel tube. Photosynthetically active radiation (PAR) was measured inside empty collection bags with sensors (S-LIA-M003) of data loggers (Hobo Micro Station, Onset Computer Corporation, Bourne, MA, USA). In weeks 22 and 26, the air temperature in the collection bags was measured using the S-THA-M006 temperature sensors of Hobo Micro Station. In weeks 31 and 36, the air temperature and relative humidity (RH) outside and inside the collection bags were monitored using wireless data loggers (Hygrochron DS1923-f5 iButton, Maxim Integrated Products, San Jose, CA, USA). In weeks 31 and 36, the bags increased the air temperature by an average of 2.8 and 1.5 °C, respectively. The RH in the bags was an average of 40%. The average air temperatures during the sampling were 23 °C (week 22), 26 °C (week 26), 34 °C (week 31) and 17 °C (week 36). The VOC compounds were analysed by GC–MS (Hewlett Packard GC type 6890, MSD 5973, Beaconsfield, UK). The compounds were desorbed (Perkin Elmer ATD400 Automatic Thermal Desorption System, Beaconsfield, UK) at 250 °C for 10 min, cryo-focused in a cold trap at −30 °C and subsequently injected onto an HP-5 capillary column (50 m × 0.2 mm i.d. ×0.33 μm film thickness, J&W Scientific, Folsom, CA, USA). The temperature programme was 40 °C for 1 min, followed by increases of 5 °C min^−1^ to 210 °C and 20 °C min^−1^ to 250 °C. The carrier gas was helium. Mass numbers from *m*/*z* 33 to 300 were recorded. The compounds were identified with the aid of authentic standards, Wiley library, and retention indexes as described in Kivimäenpää et al. (2013). The emission rates were expressed as ng g^−1^ DW h^−1^ and calculated per needle DW. Emission rates were also standardized to a temperature of +30 °C using the algorithms by Guenther et al. (1993) to make sampling occasions comparable and to study the long-term acclimation to warming by excluding the effect of momentary temperature elevation during sampling. In this paper, emission rates refer to standardized emissions unless otherwise stated.

### Gas exchange

Net photosynthesis (*P*_n_) and stomatal conductance (*g*_s_) were measured from one current- and previous-year shoot from a lateral shoot of the uppermost whorl. The measurements were taken using LiCOR 6400 XT (LI-COR Inc., Lincoln, NE, USA) with an opaque conifer chamber using subplot temperatures and a CO_2_ concentration of 400 p.p.m. and a saturating PAR level of 1000 μmol m^−2^ s^−1^, determined with a light saturation curve. Some needles were removed to fit the chamber. The detached needles were considered in the needle biomass required for the BVOC emission calculation. Gas exchange was expressed on a needle area basis. Needles were detached and photographed with a scale, and the projection area was calculated with the tools of ImageJ and converted to total needle area as done by Räsänen et al. (2012). Gas exchange data from week 31 are included in the publication of Kivimäenpää et al. (2017).

### Needle terpenoid concentrations

Two or three current- and previous-year shoots from the uppermost whorl of the seedlings were detached, weighed rapidly for their fresh weight (FW), frozen in liquid nitrogen and stored at −80 °C. The needles’ terpenoid concentrations were determined using a modification of the method described by Kainulainen et al. (1992). Two hundred milligrams of needles were ground in liquid nitrogen using a mortar and pestle, extracted for 1 h at room temperature in 2 ml of hexane containing 1-chloro-octane (36 μg ml^−1^ hexane) as internal standard and washed twice with 2 ml of n-hexane. The extracts were analysed using a gas chromatograph (6890 N, Agilent Technologies, China) equipped with a mass selective detector (type 5973 inert, Agilent Technologies, USA). Separations were carried out on a 30-metre HP-5MS 19091S-433 (i.d. 0.25 mm; film thickness 0.25 μm, Agilent J&W Scientific, USA) column. Helium was used as a carrier gas, and the linear velocity was about 40 cm s^−1^. The splitless sampling technique was used, and 2 μl was injected. The initial column temperature was 40 °C, with a hold for 1 min, and then programmed from 40 to 100 °C with a temperature increase of 5 °C min^−1^ and then to 250 °C with a temperature increase of 15 °C min^−1^ and held for 2 min. Mass numbers from *m*/*z* 30 to 300 were recorded. Compound identification and quantification were based on their mass spectra, retention time and authentic standard compounds, as described by Kainulainen et al. (1992). Concentrations were expressed both per FW and DW with the aid of the DW/FW ratio, which was determined from the needles used for gas exchange analysis. A preliminary analysis revealed the results of needle terpene concentrations calculated at FW or DW showed marked differences between the needle generations and different sampling weeks, but seldom between the treatments. The results in the main document are presented at DW (Figures 3–5), and those in the supplement are calculated at FW (Tables S3–S6, Figures S2,S3 available as Supplementary data at *Tree Physiology* Online).

### Statistics

Averages of seedlings per subplot were used in statistical analyses; thus, *n* = 3–4. The main effects of ozone (O), temperature (T) and week (W) and their 2-way and 3-way interactions on *P*_n_, *g*_s_, concentrations and emission rates of total non-oxygenated monoterpenes (nMT), oxygenated monoterpenes (oMT), non-oxygenated sesquiterpenes (nST) and oxygenated sesquiterpenes (oST) were tested by linear mixed models (LMM) analysis of variance (ANOVA). Current- and previous-year needles were tested separately (*P_n_*, *g*_s_, terpenoid concentrations). O, T and W were regarded as fixed factors and plot as a random factor. Individual extracted terpenoid compounds and volatile compounds, as well as main shoot length, shoot fractions of spruce crowns included in BVOC collection, and the proportion of seedlings with Lammas growth, were tested correspondingly, but the tests were done for each week separately. Interactions with *P*-value < 0.1 were further studied by calculating *P*-values for simple main effects (SME, i.e., post hoc test for interactions) with Bonferroni corrections. *P*-values < 0.5 of post hoc tests and main effects were considered significant. When necessary, data were logarithm or square root transformed to meet the assumptions (normality of the residuals) of LMM ANOVA. When the assumptions were not fulfilled, the treatment effect was studied with the Kruskal–Wallis test and the differences between the treatments with the Bonferroni test. Spearman correlation tests were conducted between a gas exchange (*P*_n_, *g*_s_), BVOC emission rates (total nMT, oMT, ST) and needle terpene concentrations at DW (total nMT, oMT, nST, oST), and only the significant correlations are shown in the results. All statistical analyses were performed by IBM SPSS Statistics for Windows (version 25.0, IBM Corp., Armonk, NY, USA).

Significant *P*-values of statistical tests for the total terpene concentrations at DW and emission rates of compound groups are included in the figures of the main document, but those of total terpene concentrations at FW and of individual compounds in the Supplementary files (Figures S2–S3, Tables S3–S6 available as Supplementary data at *Tree Physiology* Online for needle concentrations at FW, Tables S7–S10 available as Supplementary data at *Tree Physiology* Online for emissions). In addition, needle concentrations and emission rates of a few representative terpenoids (α-pinene, camphene, limonene, 1,8-cineole and (*E*)-β-caryophyllene) are shown in the main document. These compounds are abundant in the emission blend of Norway spruce (Kivimäenpää et al. 2013, 2021), and their emissions rates show seasonal variation (Wang et al. 2017) or respond to ozone or warming exposures (Kivimäenpää et al. 2016, Feng et al. 2019). On the other hand, the compounds differ in volatility and reactivity by oxidants in the atmosphere and abundance in the needles and stem (Table 2).

**Table 2:** Physical properties of selected terpenoids and their main storage site in Norway spruce shoots.

Compound	Chemical group	Chemical formula	Vapour pressure (kPa at 25 °C)	Atmospheric lifetime in reaction with^c^		Main storage site in shoot^d^
				OH	O_3_	
α-Pinene	nMT	C_10_H_16_	0.63^a^	2.6 h	4.6 h	Needles, bark + wood
Camphene	nMT	C_10_H_16_	0.33^b^	2.6 h	18 day	Needles
Limonene	nMT	C_10_H_16_	0.26^a^	49 min	2.0 h	Needles, bark + wood
1,8-Cineole	oMT	C_10_H_18_O	0.25^b^	1.0 day	>110 day	Needles
(*E*)-β-Caryophyllene	nST	C_15_H_24_	0.0017^a^	42 min	2 min	Needles

nMT = non-oxygenated monoterpene, oMT = oxygenated monoterpene, nST = non-oxygenated sesquiterpene. The higher the vapour pressure, the more volatile the compound.

^a^ Mofikoya et al. (2019).

^b^PubChem database (https://pubchem.ncbi.nlm.nih.gov).

^c^ Atkinson and Arey (2003).

^d^ Bufler et al. 1990, Kivimäenpää et al. (2021).

## Results

### Gas exchange

**Figure 2.**
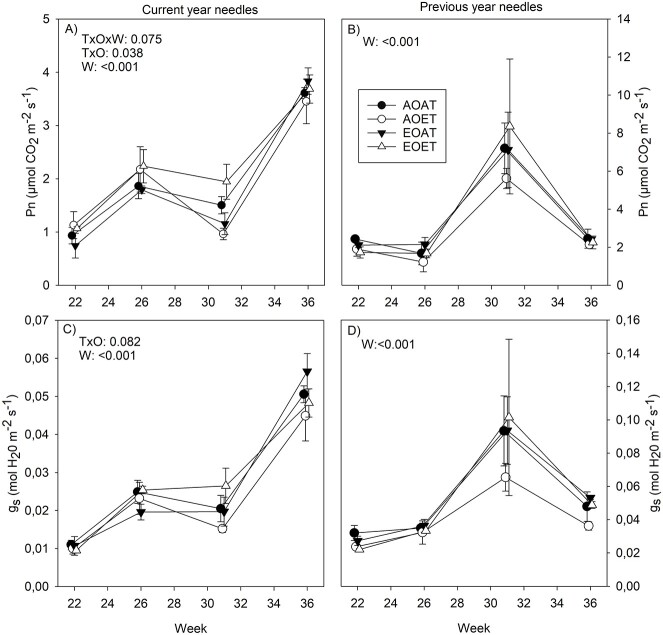
Net photosynthesis (A, B) and stomatal conductance (C, B) in current- (A, C) and previous-year (B,D) needles of Norway spruce exposed to EO and ET alone and in combination. AOAT = AO, AT; AOET = AO, ET; EOAT = EO, AT; EOET = EO, ET. Treatment averages (*n* = 3–4) with SE are shown. *P*-values for the main effects (*P* < 0.05) and interactions (*P* < 0.1) from LMM ANOVA for temperature (T), ozone (O) and week (W) are shown. Note different *y*-axis scales between current- and previous-year needles.

The net photosynthesis (*P*_n_) and stomatal conductance (*g*_s_) of the current-year needles increased from the early season to the end of the growing season (Figure 2A and C). In previous-year needles, *P*_n_ and *g*_s_ peaked in week 31 in August (Figure 2B and D). The interactive effects of ET and EO were found on *P*_n_ and *g*_s_ of current-year needles (Figure 2A and C). The interactive effect on *P*_n_ was due to reductions caused by ET and EO alone, but not in combination, in week 31 (details of pairwise comparisons in Kivimäenpää et al. 2017). Changes in other weeks were not significant. Pairwise comparisons for ozone × temperature interaction of *g*_s_ were not significant (*P* > 0.05).

### Terpene concentrations in needles

When needle terpene concentrations from all four sampling occasions, all treatments and both needle generations were averaged, 51% of total needle terpene concentrations (at DW) were calculated to consist of nMT (altogether 11 compounds), the major compounds being camphene (18% of all terpenes), α-pinene (11%), limonene (10%) and myrcene (5%). The respective proportion of total oMT (16 compounds) was 36%, the major compounds being bornyl acetate (23% of total terpene concentrations) and 1,8-cineole (5%). The proportion of nMTs was highest, and that of oMTs was consequently lower in the current-year needles in week 22 (Table S2 available as Supplementary data at *Tree Physiology* Online). The proportion of total nST (14 compounds) was 4% of total needle terpene concentration (calculated from the season, treatment and needle age average), and the respective proportion of oxygenated sesquiterpenes total oST (three compounds) was 9% (Table S2 available as Supplementary data at *Tree Physiology* Online). As an average over the season and treatments, total terpene concentration at FW was 55% higher in the previous- than the current-year needles. The respective difference in total terpene concentration at DW was 26%.

#### Monoterpenes, current-year needles

**Figure 3.**
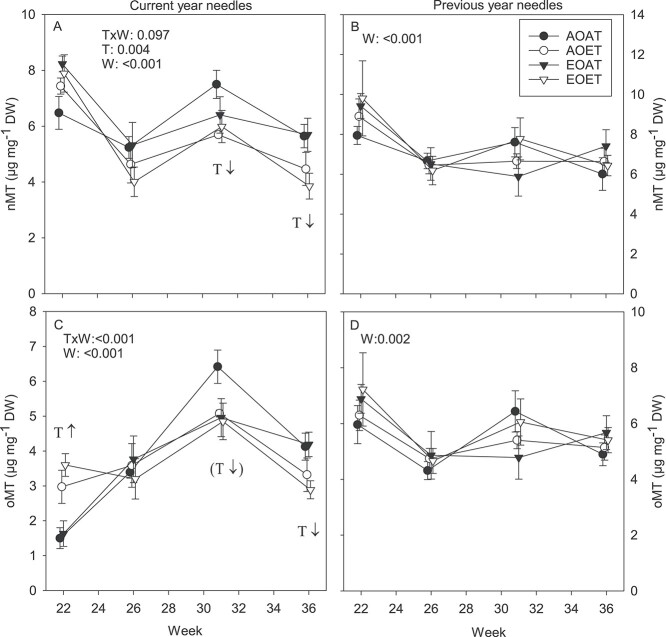
Total concentrations of non-oxygenated (A, B) and oxygenated (C, D) MTs in current- (A, C) and previous-year (B, D) needles of Norway spruce exposed to EO and ET alone and in combination in 2010. AOAT = AO, AT; AOET = AO, ET; EOAT = EO, AT; EOET = EO, ET. Treatment averages (*n* = 3–4) with SE are shown. *P*-values for the main effects (*P* < 0.05) and interactions (*P* < 0.1) from LMM ANOVA for temperature (T) and week (W) are shown. T with arrows indicates increasing ↑ or decreasing ↓ effect of warming on each week (*P* < 0.05 from SME tests of T × W interactions), marginally significant (*P* < 0.1) trends in parentheses.

Concentrations of total nMT and oMT calculated at FW (Figure S2A and C available as Supplementary data at *Tree Physiology* Online) increased from the early summer (week 22) to the beginning of August (week 31), but in September (week 36), the concentrations decreased almost to the same level as at the beginning of July (week 26). Concentrations of total oMT at DW (Figure 3C) showed similar seasonal changes as concentrations at FW. Concentrations of nMT at DW did not show the steep increase from week 22 to 31 seen in concentrations calculated at FW (Figure 3A, Figure S2A available as Supplementary data at *Tree Physiology* Online). In week 22, warming increased concentrations of total oMTs, but later during the growing season, in weeks 31 and 36, total concentrations of both nMT and oMT were lower in the ET treatments (Figure 3A and C, Figure S2A and C available as Supplementary data at *Tree Physiology* Online).

Although total nMTs were unaffected by the treatments at the beginning of the growing season (Figure 3A), warming as the main effect increased concentrations of α-pinene (Figure 5A), camphene (Figure 5B), tricyclene and terpinolene in the current-year needles (Table S3 available as Supplementary data at *Tree Physiology* Online) and reduced concentrations of limonene (Figure 5C). An analysis of individual compounds also revealed that warming-induced increase of oMT was enhanced by EO in the current-year needles in week 22, including major oMTs 1,8-cineole and bornyl acetate, as well as camphor and linalool (Figure 5D, Table S3 available as Supplementary data at *Tree Physiology* Online). Elevated ozone at AT increased myrcene concentrations in week 22 (Table S3 available as Supplementary data at *Tree Physiology* Online). On the second sampling occasion in week 26, an increasing effect of warming was noted in a few nMTs and oMTs (Table S4 available as Supplementary data at *Tree Physiology* Online). In weeks 31 and 36, concentrations of many of the major MTs were decreased by warming, including α-pinene (Figure 5A), camphene (Figure 5B), 1,8-cineole (Figure 5D), β-pinene, myrcene, sabinene and terpinolene (Tables S5 and S6 available as Supplementary data at *Tree Physiology* Online), and in week 36, the warming-induced decrease was enhanced under EO, e.g., in tricyclene and camphene at FW (Table S6 available as Supplementary data at *Tree Physiology* Online).

**Figure 4.**
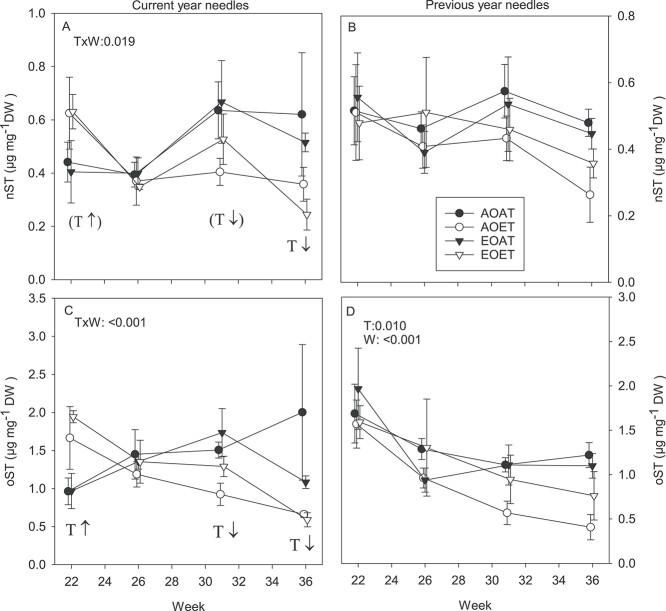
Total concentrations of non-oxygenated (A, B) and oxygenated (C, D) STs in current- (A, C) and previous-year (B, D) needles of Norway spruce exposed to EO and ET alone and in combination in 2010. AOAT = AO, AT; AOET = AO, ET; EOAT = EO, AT; EOET = EO, ET. Treatment averages (*n* = 3–4) with SE are shown. *P*-values for the main effects (*P* < 0.05) and interactions (*P* < 0.1) from LMM ANOVA for temperature (T) and week (W) are shown. T with arrows indicates increasing ↑ or decreasing ↓ effect of warming on each week (*P* < 0.05 from SME tests of T × W interactions), marginally significant (*P* < 0.1) trends in parentheses.

#### Monoterpenes, previous-year needles

Total MT concentrations increased along the growing season when calculated at FW (Figure S2B and D available as Supplementary data at *Tree Physiology* Online), whereas concentrations at DW were highest in week 22 and then decreased to a relatively constant level (Figure 3B and D). Treatment effects were not significant for total MT concentrations in previous-year needles (Figure 3B and D, Figure S2B and D available as Supplementary data at *Tree Physiology* Online). Compared with current-year needles, fewer treatment effects were observed in the concentrations of individual compounds. Elevated ozone increased concentrations of myrcene and two oMTs in week 22 (Table S3 available as Supplementary data at *Tree Physiology* Online). Decreasing effects of warming on a few compounds were reported in weeks 31 and 36, as with current-year needles (Tables S5 and S6 available as Supplementary data at *Tree Physiology* Online). In week 31, concentrations of myrcene and limonene were highest when both temperature and ozone were elevated (Table S5 available as Supplementary data at *Tree Physiology* Online).

#### Sesquiterpenes, current-year needles

**Figure 5.**
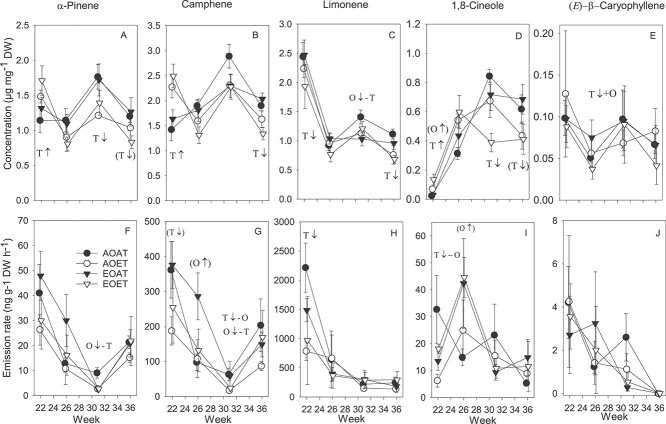
Average (*n* = 3–4) concentrations (A–E) in current-year needles and emission rates (F–J) of selected terpenoids exposed to EO and ET alone and in combination throughout a growing season 2010. AOAT = AO, AT; AOET = AO, ET; EOAT EO, AT; EOET = EO, ET. Significant (*P* < 0.05, Bonferroni test after LMM ANOVA) SMEs of interactions of ozone (O) or warming (T) are shown as increasing ↑ or decreasing ↓ effect of the one factor in the level other factors (e.g., T↓ + O = reducing effect of warming under EO, O↑ − T = increasing effect of ozone in AT). Increasing main effects with arrows are shown when interaction was not found. Marginally significant (*P* < 0.1) trends in parentheses. Error bars, SE.

Changes in total nST and oST concentrations at FW in current-year needles (Figure S3A and C available as Supplementary data at *Tree Physiology* Online) followed the same seasonal pattern as that of MTs at FW (Figure S2A and C available as Supplementary data at *Tree Physiology* Online). Warming increased total ST concentrations at the beginning of the growing season, in week 22, but decreased them in August, week 31 and September, week 36 (Figure 4A and C, Figure S3A and C available as Supplementary data at *Tree Physiology* Online). In week 22, nST compounds significantly increased by warming were β-elemene and α-muurolene, and warming-induced increases in γ-cadinene and δ-cadinene were enhanced by EO (Table S3 available as Supplementary data at *Tree Physiology* Online). In week 26, warming under EO decreased concentrations of (*E*)-β-caryophyllene at DW (Figure 5F).

#### Sesquiterpenes, previous-year needles

Concentrations of oSTs at DW decreased throughout the growing season (Figure 4D). Warming as a main effect decreased oSTs in the previous-year needles (Figure 4D, Figure S3D available as Supplementary data at *Tree Physiology* Online). The decrease by warming in nST calculated at FW (Figure S3C available as Supplementary data at *Tree Physiology* Online) was also close to significant (*P* = 0.060 for the main effect). At the compound level, the warming decreased nSTs α-muurolene, γ-cadinene and δ-cadinene in weeks 31 and 36 (Tables S5 and S6 available as Supplementary data at *Tree Physiology* Online).

### BVOC emissions

BVOC emissions consisted mostly of nMTs (altogether 14 compounds), and the proportion of their standardized emission rates was 87% of the season average of total terpenoid emissions. Respective proportions of other compound groups emitted were 4% for oMTs (11 compounds), 1% nSTs (28 compounds), 7% isoprene, < 0.5% (*Z*)-hexenyl acetate (green leaf volatile, GLV) and <0.5% methylsalicylate (benzenoid). The proportion of nMTs of total emissions was highest at the beginning of the growing season during needle and shoot growth (Table S2 available as Supplementary data at *Tree Physiology* Online). The proportion of isoprene in the emission blend was highest at the end of the growing season in August and September (Table S2 available as Supplementary data at *Tree Physiology* Online).

#### Monoterpenes

**Figure 6.**
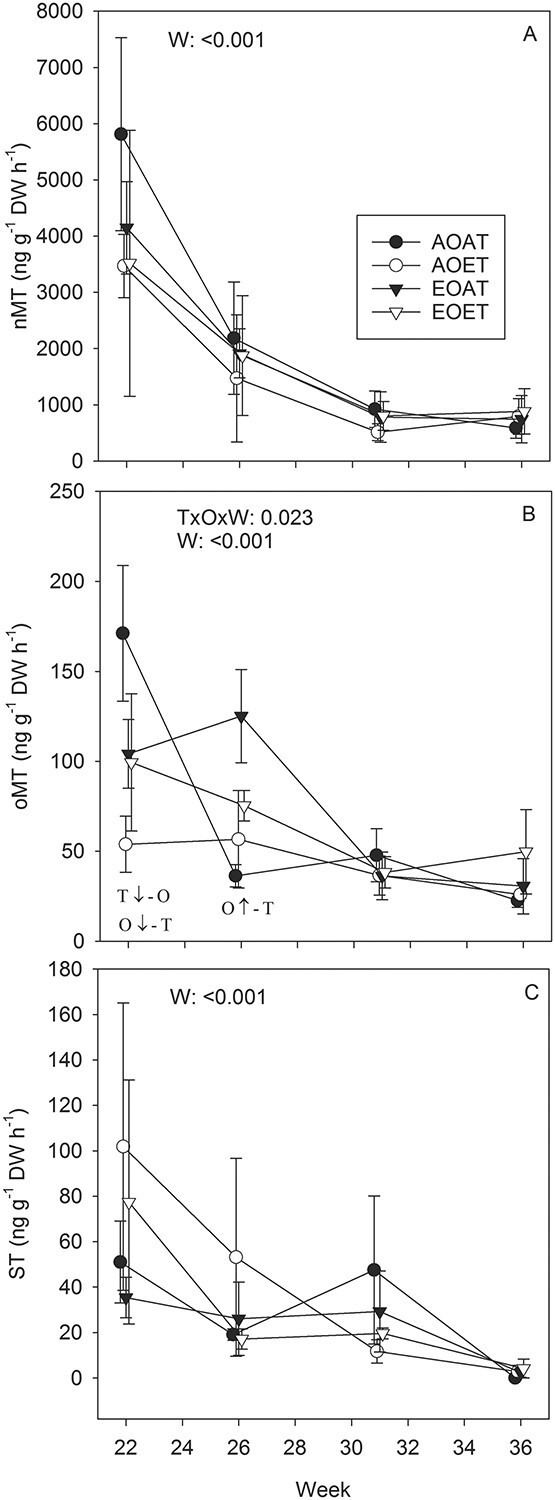
Total temperature-standardized emission rates of non-oxygenated (A) and oxygenated (B) MTs and STs (C) of Norway spruce seedlings exposed to EO and ET alone and in combination in 2010. AOAT = AO, AT; AOET = AO, ET; EOAT = EO, AT; EOET = EO, ET. Treatment averages (*n* = 3–4) with SE are shown. *P*-values for the main effects (*P* < 0.05) and interactions (*P* < 0.1) from LMM ANOVA for temperature (T), ozone (O) and week (W) are shown. Arrows indicate increasing ↑ or decreasing ↓ effect of warming or ozone in absence (−T or –O) or presence (+T or +O) of the other factor on each week (*P* < 0.05 from SME tests of T × O × W interactions).

Temperature-standardized emission rates of nMTs peaked in week 22 at the beginning of the growing season (Figure 6A). nMT emissions were 2.3 times higher in week 22 than in week 26, and 5.6 times higher than in weeks 31 and 36. The decrease in actual emission rates followed the same general pattern, but the decrease from week 22 towards the end of the growing season was less steep, with high emissions in week 31 when air temperatures were highest (Figure S4A available as Supplementary data at *Tree Physiology* Online). The nMTs significantly responding to the treatments were tricyclene, α-pinene, camphene and limonene. Warming decreased emission rates of tricyclene (Table S7 available as Supplementary data at *Tree Physiology* Online), camphene (Figure 5G) and limonene (Figure 5H) in week 22, and the decrease in actual emissions of camphene was still detectable in week 26 (Table S8 available as Supplementary data at *Tree Physiology* Online). Ozone tended to increase emissions of camphene (Figure 5G) in week 26. In week 31, standardized emissions of camphene and tricyclene were decreased by warming in AO concentrations, and EO decreased emission rates of camphene, tricyclene and α-pinene at an AT (Figure 5F and G, Table S9 available as Supplementary data at *Tree Physiology* Online). The decreases were less marked when both temperature and ozone were elevated. Actual emissions of α-pinene were increased by warming in week 31 (Table S9 available as Supplementary data at *Tree Physiology* Online).

Like nMTs, the standardized emission rates of oMTs were lower at the end of the growing season (weeks 31, 36) than at the beginning (Figure 6B). Warming and ozone had interactive effects on standardized total oMT emission rates in weeks 22 and 26 (Figure 6B). In week 22, EO at the AT and an ET at the AO concentration decreased total oMT emissions, but the decrease was less prominent when ozone and temperature were both elevated (Figure 6B). The changes in total oMTs in week 22 were due to changes in the major oMTs, 1,8-cineole (Figure 5I), camphor, borneol and bornyl acetate (Table S7 available as Supplementary data at *Tree Physiology* Online). In week 26, ozone increased total oMT emission rates at the AT (Figure 6B) and tended to increase emissions of 1,8-cineole (Figure 5G) and bornyl acetate (Table S8 available as Supplementary data at *Tree Physiology* Online). Actual emission rates of total oMTs (Figure S4B available as Supplementary data at *Tree Physiology* Online), linalool and camphor (Table S9 available as Supplementary data at *Tree Physiology* Online) were significantly increased by warming in week 31.

#### Sesquiterpenes

Treatment effects were not significant on total STs (Figure 6C, Figure S4C available as Supplementary data at *Tree Physiology* Online). Only a few STs were significantly affected by warming throughout the growing season, and the emission rates of most STs were below the detection limit in week 36 (Figure 5J, Tables S6–S10 available as Supplementary data at *Tree Physiology* Online).

#### Isoprene, GLVs and methylsalicylate

A reduction in standardized isoprene emissions by EO at the AT was observed in week 31 (Table S9 available as Supplementary data at *Tree Physiology* Online). Warming induced emissions of methylsalicylate in week 31 (Table S9 available as Supplementary data at *Tree Physiology* Online). The only GLV detected, (*Z*)-hexenyl-acetate, was unaffected by the treatments (Tables S7–S10 available as Supplementary data at *Tree Physiology* Online).

### Correlations between leaf gas exchange, BVOC emissions and needle terpenoid concentrations

#### Gas exchange and BVOC emissions

The *P*_n_ of previous-year needles was positively correlated with total nMT emissions (*r* = 0.534, *P* = 0.049, *n* = 14) and total oMT emissions (*r* = 0.538, *P* = 0.047, *n* = 14) in week 22, and the trend was the same with total ST emissions (*r* = 0.477, *P* = 0.085, *n* = 14). The *g*_s_ of current-year needles was positively correlated with total ST emissions (*r* = 0.641, *P* = 0.007, *n* = 16) in week 31.

#### Gas exchange and needle terpenoid concentrations

The *P*_n_ of previous-year needles was negatively correlated with the oMT concentrations of current-year needles (*r* = −0.613, *P* = 0.020, *n* = 14) in week 22. The *g*_s_ and nST concentration of current-year needles was positively correlated (*r* = 0.618, *P* = 0.019, *n* = 14) in week 22. In week 31, *P*_n_ and *g*_s_ were positively correlated with nMT concentrations in current-year needles (*r* = 0.562, *P* = 0.024 for *P*_n_, *r* = 0.579, *P* = 0.019 for *g*_s_, *n* = 16). In week 36, the *g*_s_ of current-year needles was positively correlated with nST concentrations in both current- (*r* = 0.514, *P* = 0.045, *n* = 15) and previous- (*r* = 0.550, *P* = 0.034, *n* = 15) year needles. The $P_{\mathrm{n}} $ of previous-year needles was also correlated with nST concentrations of previous-year needles (*r* = 0.518, *P* = 0.048, *n* = 15), and the *g*_s_ of previous-year needles was positively correlated with oST concentrations of current- and previous-year needles (*r* = 0.539, *P* = 0.038, *n* = 15 for both) in week 36.

#### BVOCs and terpenoid concentrations

The nMT emissions and nMT concentration of the previous-year needles were negatively correlated (*r* = −0.662, *P* = 0.010, *n* = 14), as were the oMT emissions and oMT concentrations of the previous-year needles (*r* = −0.547, *P* = 0.043, *n* = 14) in week 22. In week 26, nMT concentrations of current-year needles and nMT emissions correlated positively (nMT: *r* = 0.789, *P* = < 0.001, *n* = 14).

## Discussion

### Terpene needle concentrations and emissions depend on needle phenology and age

Concentrations of several nMT are low, and those of oMT can be close to zero in newly formed Norway spruce needles, but these increase rapidly during needle development (Schönwitz et al. 1990). The photosynthesis of previous-year needles is the main carbon supply for opening buds in conifers (Hansen et al. 1996 and references therein) and based on the visual phenological stage (Hall et al. 2009), new shoots (0.6–9.7 cm in length) in this study may have varyingly depended on the photosynthesis of previous-year needles. The seedlings with the most recently opened buds may explain the negative correlation between the photosynthesis of previous-year needles and the concentration of oMTs in current-year needles. The observed changes throughout the growing season in MT concentrations calculated at FW, i.e., a substantial increase from the newly developed shoots towards needle maturation at the middle and end of the growing season, and a slight decrease in the autumn, as well as similar changes of the older needles, are in line with previous studies in which concentrations were calculated at FW (Schönwitz et al. 1990 and references therein). However, when terpene concentrations were calculated at DW, and the influence of water content of the needles was reduced, seasonal changes in terpene concentrations in current-year needles were often less profound and in previous-year needles, even the opposite. The difference in terpene concentrations between needle generations was also less marked. As new information, to our knowledge, this study revealed ST concentrations (at FW) in current-year needles showed similar changes to MTs throughout the growing season and needle development and that seasonal changes also existed in previous-year needles. Positive correlations between leaf gas exchange and ST concentrations may indicate the important role of de novo ST biosynthesis in mature needles.

The seasonal pattern in the terpene emission rates of the young Norway spruce in this study accords with previous studies undertaken with mature Norway spruce individuals with high emissions from elongating needles and shoots during the spring, a co-occurrence of high emissions and hot days, and low emissions late in the growing season (Bourtsoukidis et al. 2014, van Meeningen et al. 2017, Wang et al. 2017). Terpene concentrations of current-year needles explained emission rates in June, but concentrations and emissions were not generally correlated, or their pattern was even opposite. For example, terpenoid concentration in developing shoots and needles in week 22 was the lowest throughout the season, but their emission rates were the highest at that time. This is probably explained by the fact that the thin cuticle of the growing new needles and a green stem with no bark are ineffective barriers for terpene diffusion, as also discussed by Llusià and Peñuelas (2000). Emissions from exposed resin do not explain the response because *P. abies* did not exude resin beads like, e.g., *Pinus ponderosa* in the spring (Eller et al. 2013). Furthermore, de novo MT emissions, both from the new growing shoots and previous-year needles, may affect the result, as indicated by the positive correlation between the photosynthesis of the previous-year needles and MT emissions in May. A decrease in standardized terpene emissions, especially those of nMTs from spring to June, and a stabilization in August and September seem to follow needle maturation, including thickening of the cuticle, as well as epidermis and hypodermis tissues and their cells walls during the season (Sirkka Sutinen, personal communication, Sutinen et al. 2006, Kivimäenpää et al. 2014) and highlights the influence of the developmental phase of the needles on BVOC emission rates. Epidermis thickness is known to affect the BVOC emissions of deciduous species, with a thinner epidermis leading to higher emission rates (Schollert et al. 2015, Hartikainen et al. 2020).

### Warming increased terpenoid concentrations in developing shoots but decreased them in mature needles and decreased BVOC emissions

Concentrations of MTs are low in newly formed Norway spruce needles and increase rapidly during needle and shoot development (Schönwitz et al. 1990), as discussed above. The faster development of the new shoots by warming, as indicated by earlier bud burst (Riikonen et al. 2012), and longer main shoots reported here, therefore, explain the warming-induced increase in MT concentrations (and probably the similar changes in STs) in current-year needles in the early growing season. Decreased MT emission rates by warming at the beginning of the growing season during needle and stem growth, despite the higher terpenoid concentrations, may be due to a more advanced growth phase reducing diffusion, as discussed above. Warming may have shifted the spring emission peak earlier.

The decrease in terpene concentrations of the current-year already mature needles by warming in August and September may have several explanations. First, the seasonal decrease in terpene concentrations took place earlier in warming treatments due to advanced phenology, as reported for the autumn of 2009 and spring of 2010 (Riikonen et al. 2012). Second, the change resulted from an indirect effect of increased nitrogen (N) uptake by warming, which increased the needle N concentrations of this study’s seedlings (Kivimäenpää et al. 2017). Kainulainen et al. (1996) have shown that increased N availability in soil decrease MT concentrations, especially in mature needles. Third, the Lammas growth apparent at the end of the season, possibly due to improved N status (Landis 2012), affected terpene concentrations of current-year needles in the warming treatments. Low terpenoid concentrations in the needles of developing shoots coincide with lower concentrations in the previous-year shoots (Schönwitz et al. 1990). There may be a similar relationship between the needle generations of the new Lammas shoots and the current shoots at the end of the season. Fourth, long-term acclimation to warming that caused a reduction in resin duct size in these seedlings (Kivimäenpää et al. 2017) may indicate reduced space for terpene storage in the needles, resulting in reduced terpenoid concentrations (Björkman et al. 1998). Warming also reduced oSTs in the previous-year needle generation, but a change in resin duct size is not a likely explanation because needle anatomy is largely determined in the buds, and the buds (formed 2008) of the older needles were formed before exposure to environmental conditions. Instead, oST synthesis may have been reduced by the warming treatment, in the same way as a 10 °C temperature elevation decreased the biosynthesis of nerolidol (oST) in the needles of Douglas fir (*Pseudotsuga menziesii*) (Duan et al. 2019).

Our results may imply that a temperature increase of only 1.1–1.3 °C can reduce terpenoid concentrations in the mature needles of young spruce. These long-term warming responses in the field contrast with the increased MT concentrations in mature needles of the same species in response to a +4 °C temperature elevation in a chamber exposure (50 days) study by Sallas et al. (2003), highlighting the effect of experimental conditions.

Warming changed the BVOC profile to a higher proportion of oMTs in June during the experiment’s first year (Kivimäenpää et al. 2013), but this study shows that the standardized emissions of many compounds were mainly decreased by warming in AO concentration in the second year of the experiment, which was supported by reduced terpenoid concentrations in matured needles. The differences between years may indicate cumulative stress effects by long-term warming on the seedlings of Norway spruce, a species adapted to shady, moist and cool growing conditions (Caudullo et al. 2016). Warming increases evapotranspiration, but the influence of soil drought in the second growing season is unlikely because the soil was kept well-watered, and the stomatal conductance was unaltered. The observed decreases in BVOC emissions by warming in Norway spruce contrasts strongly with increased BVOC emissions by other boreal tree species, *Pinus sylvestris*, *Betula pendula* and *Populus tremula*, exposed to a similar temperature increase and experimental setup (Hartikainen et al. 2009, 2012, Kivimäenpää et al. 2016). Higher actual emission rates of α-pinene, linalool and camphor in August by warming appeared to be the result of the momentary heating effect of the IR lamps instead of acclimation to long-term warming, because the temperature-standardized emissions of these compounds were unaffected by warming. Induced methylsalicylate emission by warming in August could partly be caused by aphids in some spruces because aphids are known to induce methylsalicylate emissions from conifer seedlings (Mentel et al. 2013, Kivimäenpää et al. 2020).

### Elevated ozone increased concentrations of less volatile terpenoids and emissions of less reactive BVOCs and interacted with warming

Previous experiments with EO in growth chambers, open-top chambers or open-field-conditions have reported no effects (Bufler et al. 1990, Kainulainen et al. 1995), reductions (Heller et al. 1990) or increases (Kainulainen et al. 2000) in MT or ST compound concentrations in needles of young Norway spruce. A common feature of these studies is that mature needles were studied. Our study showed that EO in combination with ET could increase concentrations of several oxygenated MT compounds as well as a few STs in the actively growing shoots. In addition, ozone could increase MT concentrations in the older, mature needles at the beginning of the growing season. The enhancing effect of ozone on warming responses in the developing shoots may indicate that faster development by warming increased the capacity of ozone-exposed needles to synthesize terpenoids to protect new shoots from high ozone concentrations typical of the spring and early growing season. The seedlings may have been responding by increased synthesis of oMTs (e.g., 1,8-cineole, linalool, camphor, bornyl acetate) and SQTs (e.g., γ-cadinene, δ-cadinene), which are lower volatility terpenoids (Mofikoya et al. 2019), to maintain the defensive potential in conditions with higher oxidative stress and higher terpenoid volatility due to warming. At the end of the growing season, the decrease in the concentrations of a few compounds such as camphene and tricyclene was strongest when EO and warming were combined. This may imply a reduction in plant chemical defence as a response to accumulated multiple stresses towards the end of the season and may have contributed to the observed cellular damage (degenerating or collapsed cells, condensation of cytoplasm) in the needles by elevated warming and ozone together in December in this study (Kivimäenpää et al. 2014).

Warming and EO modified each other's effects in terpenoid emissions, and the common response was that a decrease by one factor was less marked than a decrease by the other. For example, decreasing effects of warming on oMT emissions at the beginning of the growing season were observed only at an AO level and could be partly explained by higher oMT concentrations when warming and EO was combined. Another example is that emissions of isoprene, tricyclene, α-pinene and camphene were decreased by ozone or warming alone, but not in combination in August. One could speculate that the observed interactions in BVOC emissions were indicative of a higher need for volatile terpenoid-based defence against multiple stresses. In May, developing shoots need to be protected to ensure successful growth and bud formation. In conditions with both a high temperature and high ozone concentrations, as in our study in August, BVOCs could quickly respond to high stress by quenching ozone or ROS, stabilizing membranes sensitive to high temperatures and high ozone concentrations or activating the other defence mechanisms against oxidative stress (Velikova et al. 2005, Loreto and Schnitzler 2010, Monson et al. 2021).

Ozone doubled MT (mainly non-oxygenated) emissions in June, but not in August, during the first exposure year of this study (Kivimäenpää et al. 2013). Kivimäenpää et al. (2013) discussed whether BVOCs responded only to sufficiently high ozone concentration or if the BVOCs responded in a hormetic dose manner, being increased by the small ozone dose at the beginning of the exposure and decreased when the dose increased. Here, in the study’s second year, ozone concentrations were higher than in the first year, and AOT40 exceeded the current critical level to protect forest trees from the ozone of 5 p.p.m.h (Mills et al. 2017). Ozone increased oMT in June but reduced nMT compounds in August. The results of the second year support a hypothesis of a hormetic dose–response of BVOCs to long-term chronic ozone exposure, but also suggest that spruce seedlings modify their BVOC emissions towards oxygenated and less reactive compounds under longer-term exposure.

### Implications for plant defence and atmospheric chemistry

Long-term acclimation to warming and EO affected the terpene-based chemistry of Norway spruce needles. An increase in air temperature of only 1 °C can reduce terpenoid concentrations and defence in younger and older needle generations of young spruce. The defence potential of seedlings may be lowest at the end of the growing season in future warmer conditions. This may increase the sensitivity of seedlings against herbivory and fungal diseases, which are predicted to increase because of climate warming (La Porta et al. 2008, Jactel et al. 2019, Venäläinen et al. 2020). On the other hand, the sensitive growing shoots seemed better protected against combined warming and EO, with higher oMT concentrations. The EO concentrations and AOT40 level of this study are realistic and typical in many Central European regions where Norway spruce grows (Hjellbrekke and Solberg 2019, 2020).

From the perspective of climate change modelling, the parts of plants where the altered BVOC emissions originate are noteworthy. Considering the differences in terpenoid profile between needles and stems of Norway spruce (Bufler et al. 1990, Kivimäenpää et al. 2021, Table 2), the results of this study suggest that both warming and EO mostly affected emissions from needles, even if the woody parts of the coniferous trees could contribute substantially to tree BVOC emissions (Šimpraga et al. 2019). For example, oMTs in general and the nMTs α-pinene, camphene, limonene and tricyclene affected by warming and ozone in this study, are abundant in both younger and older needles, but lesser constituents in the woody parts of Norway spruce (Bufler et al. 1990, Kivimäenpää et al. 2021), whereas terpenes abundant in the spruce stem, such as β-phellandrene and 3-carene (Bufler et al. 1990, Kivimäenpää et al. 2021), were unaffected by the treatments.

This study of young spruce supports the previous studies done mainly with individual large coniferous trees, emphasizing the significance of the time of the growing season and the developmental phase of the shoots and needles for the BVOC emissions that influence SOA formation (Bourtsoukidis et al. 2014, Taipale et al. 2020). Long-term acclimation to warming may either shift the spring emission peak earlier or reduce the emissions from the growing shoots. Long-term acclimation to warming can also reduce emissions from mature needles of Norway spruce. This suggests that the acclimation of spruce BVOC emissions to warming also decreases the oxidative capacity of the atmosphere, in contrast to the increased BVOC emissions of other boreal trees, *P. sylvestris*, *B. pendula* and *P. tremula*, in response to warming (Hartikainen et al. 2009, 2012, Kivimäenpää et al. 2016). Tree species composition in forests in future climatic conditions will therefore also affect BVOC emissions, assuming the young and old trees acclimate to warming in the same way. The effects of spruce BVOC emissions under EO may be minor on atmospheric chemistry, because most of the compounds increased by ozone have a relatively long lifetime in the atmosphere (Atkinson and Arey 2003) and are minor compounds in the BVOC blend.

## Conclusions

The study showed that moderately ET and EO affect seasonal changes in the terpenoid concentrations and emissions of young Norway spruce, but their impact on the emissions was smaller than the seasonal variation. Climate warming can reduce the terpenoid-based defence of spruce, but the growing shoots may be well protected against an ET and increased ozone concentrations. Climate warming or EO may not increase the oxidative capacity of the atmosphere in spruce-dominated areas.

## Supplementary Material

supplement_Effects_of_elevated_ozone_and_warming_on_tpac019
