# Correction to: Effects of elevated ozone and warming on terpenoid emissions and concentrations of Norway spruce depend on needle phenology and age

**DOI:** 10.1093/treephys/tpac044

**Published:** 2022-04-13

This is a correction to: Minna Kivimäenpää, Johanna Riikonen, Hanna Valolahti, Häikiö Elina, K Jarmo Holopainen, Toini Holopainen, Effects of elevated ozone and warming on terpenoid emissions and concentrations of Norway spruce depend on needle phenology and age, *Tree Physiology*, 2022, tpac019, https://doi.org/10.1093/treephys/tpac019.

In the originally published version of this manuscript, the fifth author's name was ordered incorrectly. Also, a key and the text “<0.001” were missing from Figure 2B.

The fifth author's name should read “Jarmo K. Holopainen” instead of “K. Jarmo Holopainen”.

These errors have been corrected.

