# Supplementary material for: Effects of elevated ozone and warming on terpenoid emissions and concentrations of Norway spruce depend on needle phenology and age
Source: Tree Physiol. 2022 Feb 19;42(8):1570–86. doi: 10.1093/treephys/tpac019 (PMC9366870; doi:10.1093/treephys/tpac019)
Supplement: supplement_Effects_of_elevated_ozone_and_warming_on_tpac019 [file supplement_effects_of_elevated_ozone_and_warming_on_tpac019.pdf]

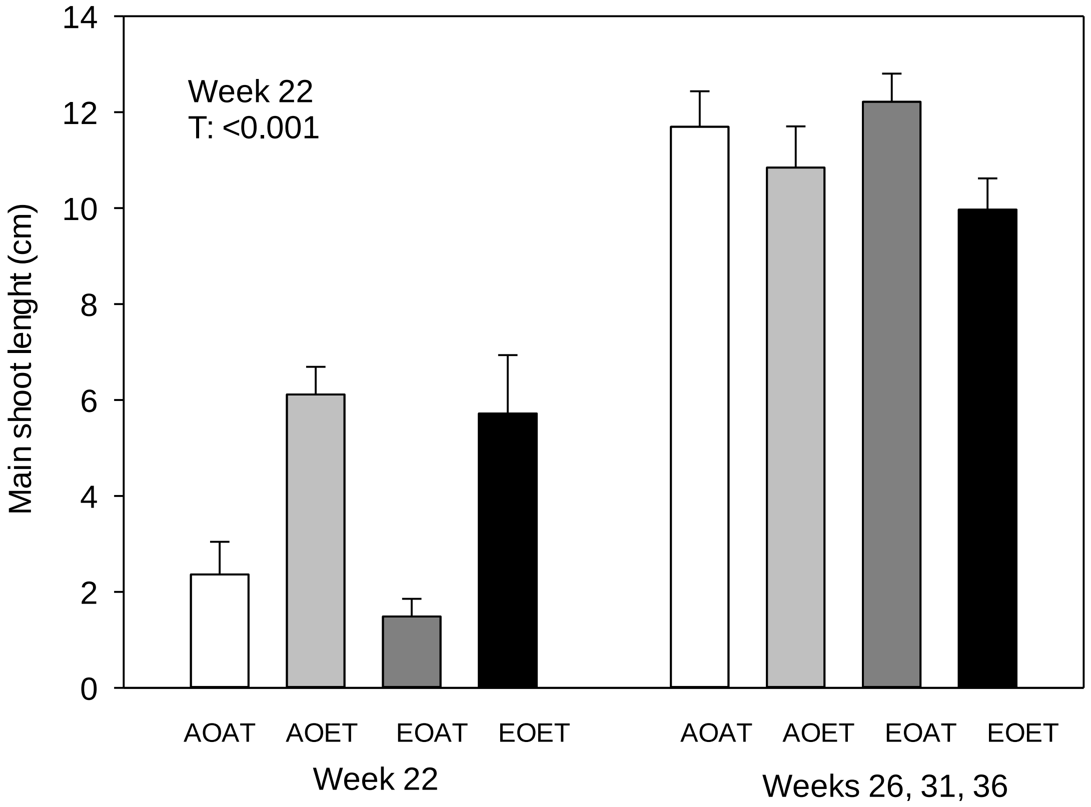

Supplementary Figure 1. Length of current year's main shoot of Norway spruce seedlings exposed to elevated ozone and elevated temperature alone and in combination in 2010. AOAT = ambient ozone, ambient temperature, AOET = ambient ozone, elevated temperature, EOAT = elevated ozone, ambient temperature, EOET = elevated ozone, elevated temperature. Bars show averages + SE on week 22 when shoots were growing (*n*=8) and on weeks 26, 31 and 36 (*n*=24, all time points pooled) when growth had ended. Significant main effect of warming (T) on week 22 is shown (Linear Mixed Models ANOVA).

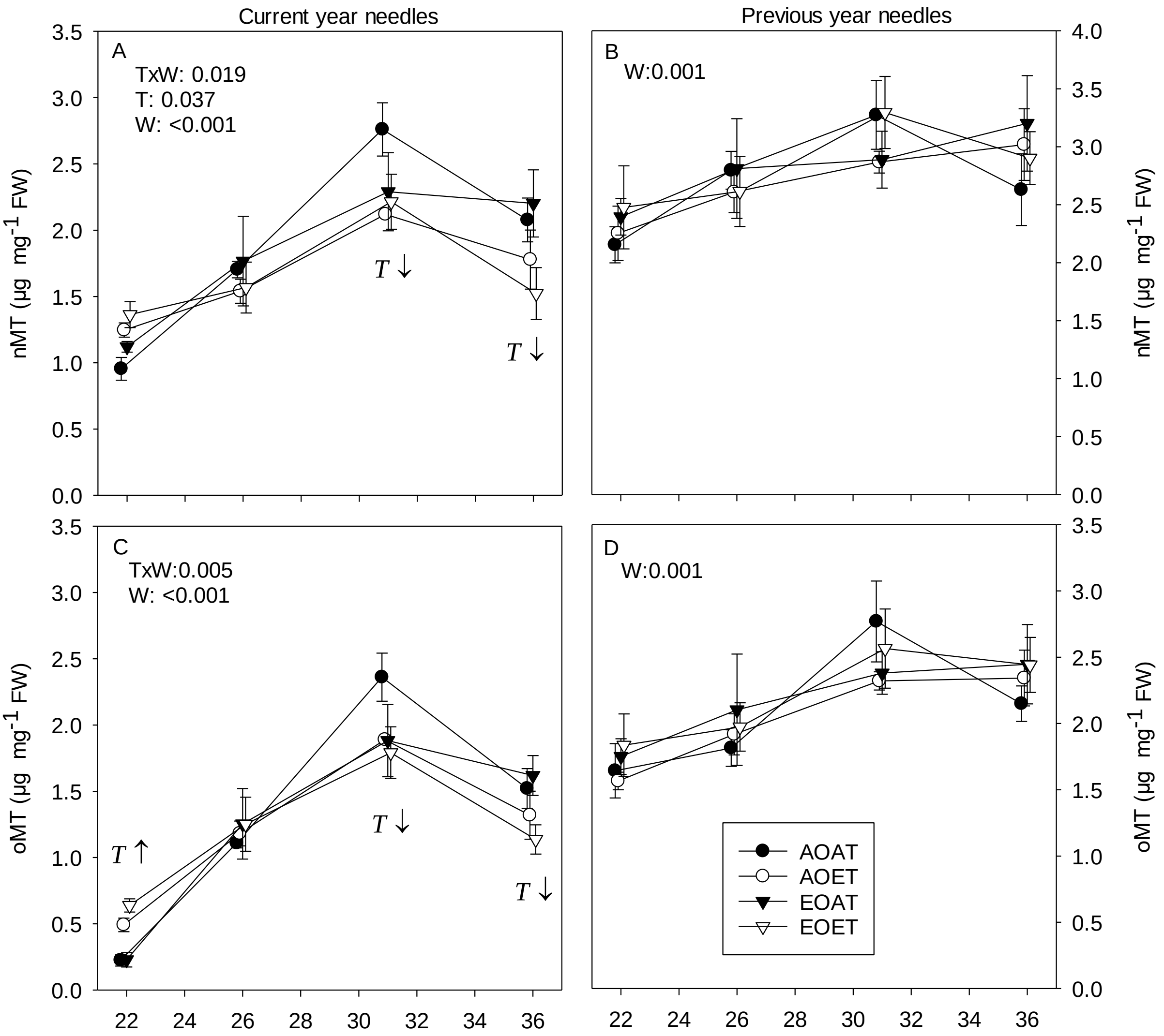

Supplementary Figure 2. Total concentrations (at FW) of non-oxygenated (A, B) and oxygenated (C, D) monoterpenes in current (A, C) and previous year (B, D) needles of Norway spruce exposed to elevated ozone and elevated temperature alone and in combination in 2010. AOAT = ambient ozone, ambient temperature, AOET = ambient ozone, elevated temperature, EOAT = elevated ozone, ambient temperature, EOET = elevated ozone, elevated temperature. Treatment averages (*n*=3-4) with SE are shown. *P*-values for the main effects ($P<0.05$) and interactions ($P<0.1$) from Linear Mixed Models ANOVA for temperature (T) and week (W) are shown. T with arrows indicate increasing ↑ or decreasing ↓ effect of warming on each week (P <0.05 from simple main effect tests of TxW interactions).

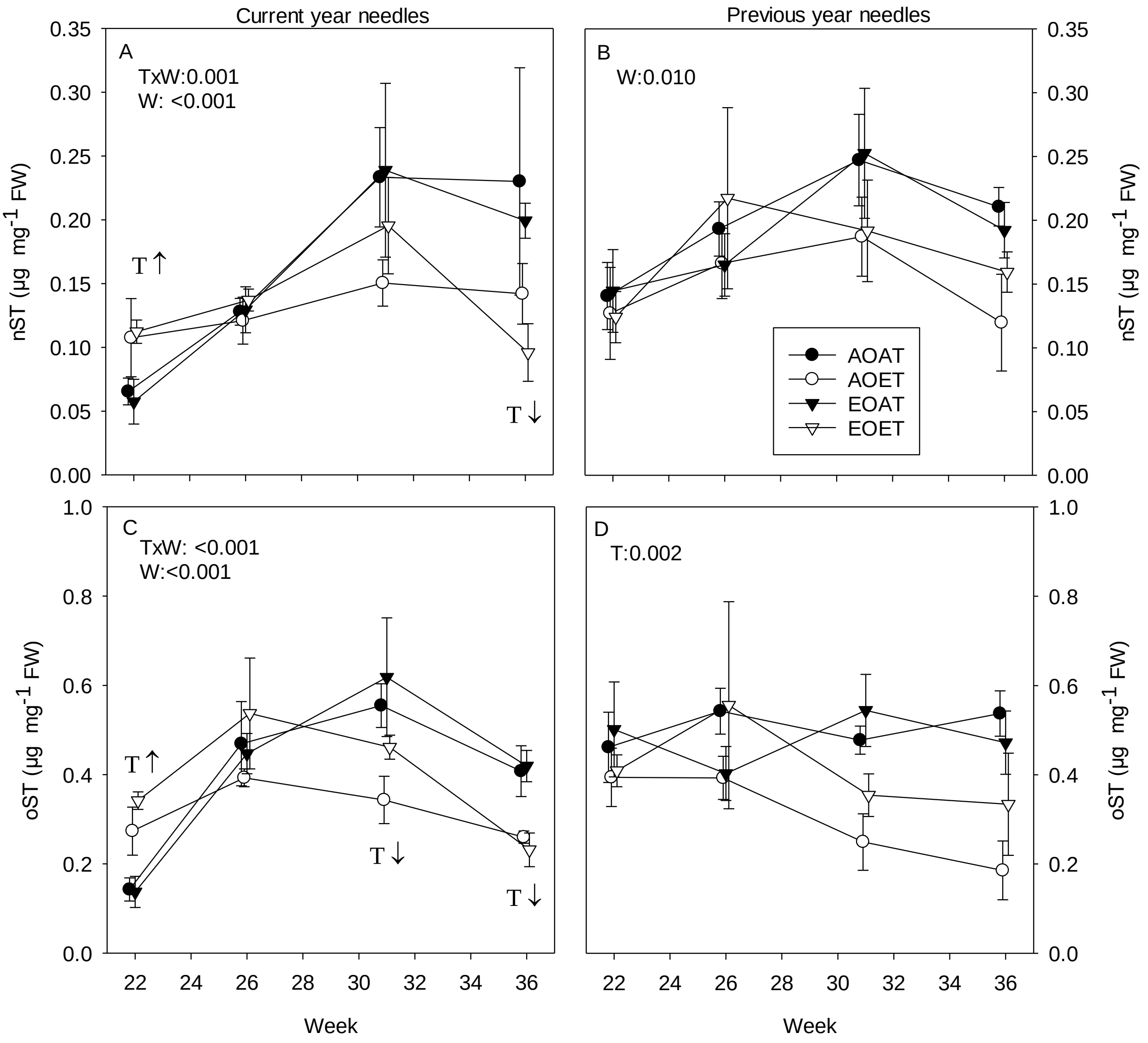

Supplementary Figure 3. Total concentrations (at FW) of non-oxygenated (A, B) and oxygenated (C, D) sesquiterpenes in current (A, C) and previous year (B, D) needles of Norway spruce exposed to elevated ozone and elevated temperature alone and in combination in 2010. AOAT = ambient ozone, ambient temperature, AOET = ambient ozone, elevated temperature, EOAT = elevated ozone, ambient temperature, EOET = elevated ozone, elevated temperature. Treatment averages (n=3-4) with SE are shown. P-values for the main effects (P<0.05) and interactions (P<0.1) from Linear Mixed Models ANOVA for temperature (T) and week (W) are shown. T with arrows indicate increasing ↑ or decreasing ↓ effect of warming on each week (P <0.05 from simple main effect tests of TxW interactions).

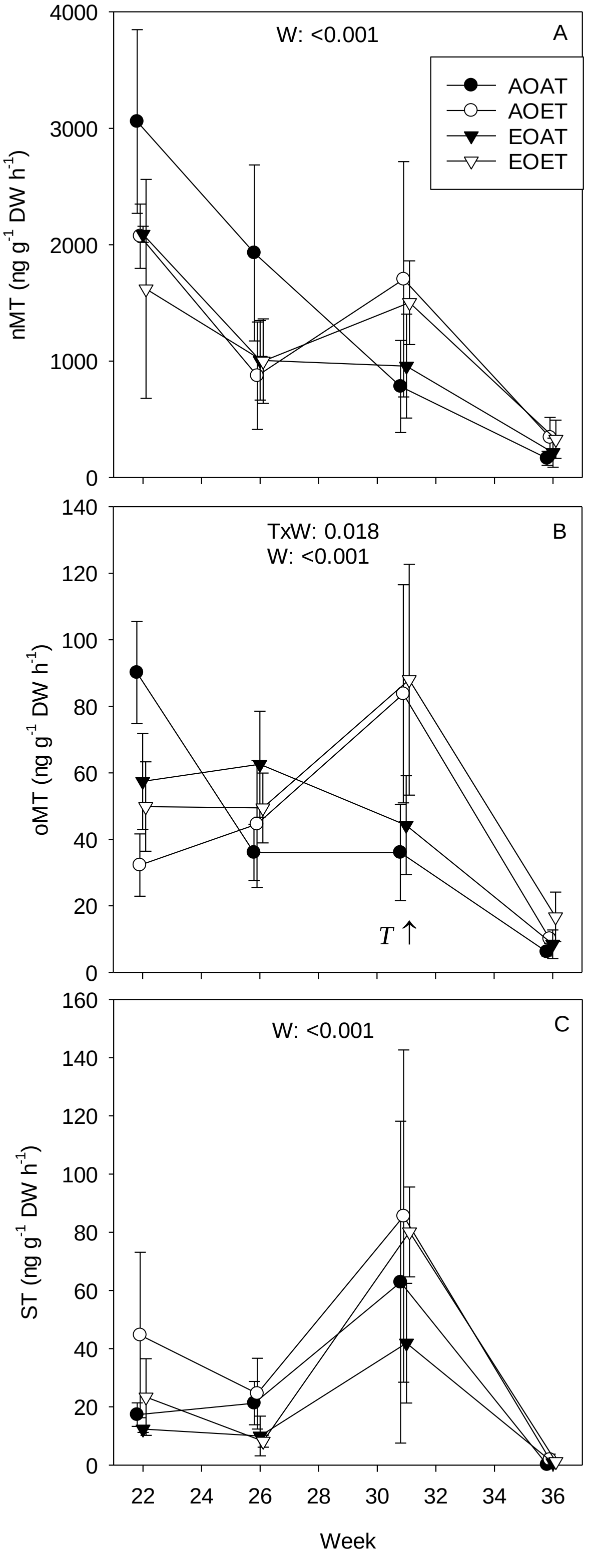

Supplementary Figure 4. Actual total emission rates of non-oxygenated (A) and oxygenated (B) monoterpenes and sesquiterpenes (C) of Norway spruce seedlings exposed to elevated ozone and elevated temperature alone and in combination in 2010. AOAT = ambient ozone, ambient temperature, AOET = ambient ozone, elevated temperature, EOAT = elevated ozone, ambient temperature, EOET = elevated ozone, ambient temperature. Treatment averages (*n*=3-4) with SE are shown. P-values for the main effects (P<0.05) and interactions (P<0.1) from Linear Mixed Models ANOVA for temperature (T) and week (W) are shown. Arrow indicate increasing ↑ effect of warming on the week, when *P* <0.05 for T from simple main effect tests of TxW interactions.

Supplementary Table 1. Average proportions (SE, *n*=3-4) of shoot fractions of spruce crowns included in BVOC collections, and proportion of seedlings with lammas growth, in the treatments AOAT = ambient ozone, ambient temperature, AOET = ambient ozone, elevated temperature, EOAT = elevated ozone, ambient temperature, EOET = elevated ozone, elevated temperature in 2010. *P*-values from Linear Mixed Models ANOVA for the main effect (<0.05) of warming (T) and ozone (O) and their interaction (<0.1) are shown, ns = not significant.

	Week 22	Week 26	Week 31	Week 36
Needles of total DW %				
AOAT	62 (2)	60 (1)	52 (<0.5)	49 (1)
AOET	65 (1)	62 (1)	54 (1)	48 (1)
EOAT	57 (1)	64 (2)	53 (1)	47 (1)
EOET	70 (1)	61 (1)	50 (2)	45 (1)
T	0.001	ns	ns	0.022
O	ns	ns	ns	ns
T x O	0.024	ns	0.099	ns
Current year needles of total needle DW %				
AOAT	43 (2)*	70 (2)	69 (2)	67 (2)
AOET	63 (<0.5)*	70 (3)	58 (2)	63 (1)
EOAT	39 (1)*	71 (2)	67 (2)	69 (2)
EOET	62 (3)*	71 (1)	62 (5)	65 (<0.5)
T	<0.001	ns	0.007	0.028
O	ns	ns	ns	ns
T x O	ns	ns	ns	ns
Seedlings with lammas growth %				
AOAT	0 (0)	0 (0)	3 (1)	2 (1)
AOET	0 (0)	0 (0)	11 (2)	10 (<0.5)
EOAT	0 (0)	0 (0)	4 (2)	4 (1)
EOET	0 (0)	0 (0)	11 (4)	8 (2)
T	-	-	0.010	0.001
O	-	-	ns	ns
T x O	-	-	ns	ns
Current year growth (needles + stems) of total DW %				
AOAT	26 (2)	54 (3)	52 (1)	49 (1)
AOET	41 (1)	56 (2)	48 (2)	46 (1)
EOAT	22 (1)	57 (2)	51 (2)	50 (2)
EOET	44 (2)	57 (2)	48 (3)	45 (1)
T	<0.001	ns	ns	0.020
O	ns	ns	ns	ns
T x O	0.085	ns	ns	ns
Older growth (needles + stems) of total DW %				
AOAT	74 (2)	46 (3)	46 (<0.5)	49 (1)
AOET	59 (1)	44 (2)	41 (1)	44 (1)
EOAT	78 (1)	43 (2)	45 (2)	46 (2)
EOET	56 (2)	43 (2)	45 (3)	47 (2)
T	<0.001	ns	ns	ns
O	ns	ns	ns	ns
T x O	0.085	ns	ns	0.073

*Growing flesh stem included in needle biomass.

Supplementary Table 2. Average (SE) proportions (%) of terpenoid classes of total needle terpenoid concentrations[a] in current and previous year needles and proportions of BVOC classes of total temperature-standardized BVOC emission rates averaged over all treatments during the growing season 2010.

	Week 22	Week 26	Week 31	Week 36
Current needles				
nMT	65 (2)	48 (1)	47 (<0.5)	49 (1)
oMT	19 (2)	35 (1)	39 (1)	36 (1)
nSQT	4 (<0.5)	4 (<0.5)	4 (<0.5)	4 (<0.5)
oSQT	11 (1)	13 (1)	10 (1)	10 (1)
Previous needles				
nMT	51 (1)	51 (1)	50 (<0.5)	50 (1)
oMT	37 (1)	37 (1)	40 (1)	40 (1)
nSQT	3 (<0.5)	3 (<0.5)	4 (<0.5)	3 (<0.5)
oSQT	10 (1)	9 (1)	7 (1)	7 (1)
BVOC				
isoprene	1 (<0.5)	1 (<0.5)	10 (4)	15 (7)
nMT	95 (1)	91 (1)	81 (4)	81 (7)
oMT	3 (<0.5)	5 (1)	6 (1)	4 (1)
nSQT	1 (<0.5)	2 (<0.5)	3 (1)	<0.5 (0.5)
[b]other	0 (0)	1 (1)	1 (<0.5)	0 (0)

Abbreviations nMT=non-oxygenated monoterpenes, oMT = oxygenated monoterpenes, nSQT= non-oxygenated sesquiterpenes, oSQT= oxygenated sesquiterpenes, BVOC = Biogenic organic volatile compound

[a] Concentrations calculated on DW

[b] Methylsalicylate + (*Z*)-3-hexenyl-acetate

Supplementary table 3. Terpene concentrations (µg/g FW) of current and previous year needles of Norway spruce seedlings exposed to elevated ozone and elevated temperature alone or in combination on week 22 2010. Compounds are listed in order of their retention time. Non-oxygenated monoterpenes in normal font, *oxygenated monoterpenes* in *cursive*, **non-oxygenated sesquiterpenes (nST) emboldened**, ***oxygenated sesuiterpenes (oST) emboldened, cursive***. AOAT = ambient ozone, ambient temperature, AOET = ambient ozone, elevate temperature, EOAT = elevated ozone, ambient temperature, EOET = elevated ozone, elevated temperature. Values are means (SE) of four treatment replicates. P-values < 0.05 for the main effects of warming (T) and ozone (O) and P-values <0.1 for the TxO interaction from Mixed Models ANOVA are shown. Significant (*P*<0.05, Bonferroni test) simple main effects of interactions explained in parentheses (e.g., T↓+O = warming reduces under elevated ozone, O↑-T = elevated ozone increases in ambient temperature). Increasing ↑ and decreasing ↓ main effects shown. Tr: *P*-values from Kruskal-Wallis test (differences between treatments, when *P*<0.05 for Bonferroni test).

	Current year needles					Previous year needles				
Compound	AOAT	AOET	EOAT	EOET	*P*	AOAT	AOET	EOAT	EOET	*P*
Tricyclene	19 (4)	37 (1)	20 (3)	49 (5)	T:0.001 ↑	115 (33)	84 (12)	82 (7)	101 (18)	
α-Pinene	168 (26)	248 (6)	181 (22)	299 (34)	T:0.008↑	493 (55)	481 (51)	497 (56)	584 (116)	
Camphene	210 (33)	377 (14)	227 (29)	441 (38)	T:0.001 ↑	666 (71)	812 (82)	777 (64)	900 (136)	
Sabinene	0 (0)	1 (1)	1 (1)	2 (2)		76 (14)	53 (15)	97 (7)	74 (15)	
β-Pinene	69 (15)	63 (16)	94 (8)	58 (9)		116 (34)	89 (10)	118 (17)	98 (14)	
Myrcene	113 (12)	121 (7)	235 (35)	160 (16)	T:0.002, TxO: <0.001 (T↓+O, O↑-T)	242 (18)	218 (7)	309 (17)	277 (44)	O:0.049↑
α-Phellandrene	3 (<0.5)	4 (<0.5)	3 (1)	6 (<0.5)	T:<0.001, O:0.006, TxO:0.042 (T:↑+O, O↑+T)	7 (1)	6 (2)	9 (2)	6 (2)	
3-Carene	5 (1)	4 (1)	16 (7)	9 (5)		15 (6)	33 (19)	34 (13)	25 (19)	
Limonene	358 (35)	378 (44)	333 (32)	325 (55)		400 (39)	459 (116)	443 (26)	387 (26)	
1,8-Cineole	4 (1)	12 (1)	4 (1)	24 (6)	T:0.001, O:0.049, TxO:0.069 (O↑+T, T↑+O)	245 (55)	165 (44)	315 (27)	246 (43)	
(*E*)-β-Ocimene	0 (0)	0 (0)	0 (0)	0 (0)		3 (2)	0 (0)	5 (3)	9 (5)	
(E)-β-Sabinene hydrate	0 (0)	0 (0)	0 (0)	0 (0)		11 (4)	5 (3)	15 (2)	14 (5)	
Terpinolene	9 (1)	14 (1)	11 (1)	17 (2)	T:0.008↑	21 (2)	17 (3)	26 (2)	15 (5)	T: 0.047↓
Linalool	<0.5 (<0.5)	1 (<0.5)	<0.5 (<0.5)	3 (1)	Tr:0.010 (EOET>AOAT, EOET>EOAT)	20 (3)	15 (2)	19 (2)	21 (3)	
Camphor	<0.5 (<0.5)	2 (1)	0 (0)	2 (<0.5)	Tr:0.021 (EOET>EOAT)	98 (46)	45 (5)	38 (13)	44 (7)	
Exomethyl camphenilol	0 (0)	18 (12)	1 (1)	20 (10)		67 (27)	86 (38)	77 (11)	100 (26)	
Borneol	4 (1)	12 (2)	2 (1)	17 (8)	Tr:0.022	147 (29)	107 (22)	80 (24)	52 (11)	
α-Terpineol	1 (<0.5)	3 (<0.5)	1 (<0.5)	9 (3)	Tr:0.015 (EOET>AOAT)	107 (20)	67 (15)	114 (13)	116 (27)	
cis-Piperitol	0 (0)	0 (0)	0 (0)	1 (1)		0 (0)	0 (0)	0 (0)	1 (1)	

Supplementary table 3 continues

	Current year needles					Previous year needles				
Compound	AOAT	AOET	EOAT	EOET	*P*	AOAT	AOET	EOAT	EOET	*P*
Berbenone	0 (0)	0 (0)	0 (0)	0 (0)		2 (1)	2 (2)	0 (0)	3 (2)	
α-Fenchylacetate	0 (0)	1 (1)	0 (0)	1 (1)		3 (1)	2 (2)	5 (2)	10 (2)	O:0.025↑
Citronellol	0 (0)	0 (0)	0 (0)	0 (0)		11 (5)	16 (9)	10 (4)	14 (6)	
Piperitone	0 (0)	0 (0)	0 (0)	2 (2)		4 (4)	6 (6)	14 (9)	14 (14)	
Bornylacetate	216 (41)	440 (40)	219 (54)	550 (41)	T:<0.001, TxO:0.061 (T↑-O, T↑+O)	888 (121)	1027 (58)	982 (50)	1157 (139)	
Exo-2-hydroxy cineoleacetate	0 (0)	1 (1)	1 (1)	2 (2)		10 (4)	11 (6)	23 (5)	14 (2)	
unknown oMT	0 (0)	2 (2)	0 (0)	5 (1)	Tr:0.018 (EOET>AOAT, EOET>EOAT)	22 (8)	8 (5)	54 (14)	24 (1)	T:0.041↓ O:0.031↑
**Longipinene**	3 (1)	2 (2)	5 (2)	2 (2)		8 (2)	5 (3)	7 (3)	4 (4)	
Geranylacetate	0 (0)	0 (0)	0 (0)	3 (3)		8 (1)	4 (2)	6 (3)	7 (4)	
**β-Elemene**	2 (1)	6 (1)	2 (1)	8 (1)	T:0.002↑	19 (2)	18 (3)	18 (3)	15 (1)	
**Longifolene**	3 (1)	3 (1)	4 (1)	5 (3)		8 (1)	6 (1)	9 (1)	12 (5)	
**β-Caryophyllene**	15 (3)	23 (15)	14 (4)	16 (5)		16 (3)	18 (9)	16 (4)	18 (8)	
**unknown nST**	1 (1)	1 (1)	1 (1)	0 (0)		3 (2)	2 (2)	1 (1)	0 (0)	
**(*E*)-β-farnesene**	1 (<0.5)	1 (<0.5)	1 (<0.5)	2 (1)		6 (1)	6 (1)	5 (<0.5)	7 (3)	
**β-Cubebene**	0 (0)	0 (0)	0 (0)	0 (0)		3 (2)	1 (1)	2 (1)	4 (2)	TxO: 0.066
**α-Humulene**	10 (2)	17 (11)	10 (3)	9 (1)		12 (2)	14 (7)	12 (3)	10 (3)	
**α-Amorphene**	0 (0)	0 (0)	0 (0)	0 (0)		1 (1)	0 (0)	2 (1)	5 (1)	O:0.045 TxO:0.076 (O↑+T)
**Germacrene-D**	17 (3)	22 (10)	12 (3)	22 (7)		22 (3)	19 (8)	19 (3)	18 (3)	
**α-Muurolene**	2 (1)	11 (3)	3 (2)	13 (1)	T:0.003↑	17 (4)	10 (3)	16 (6)	11 (5)	
**γ-Cadinene**	6 (2)	11 (3)	3 (2)	18 (<0.5)	T:0.004, TxO:0.019 (T↑+O, O↑+T)	12 (3)	13 (4)	19 (7)	11 (3)	
**δ-Cadinene**	6 (2)	19 (2)	3 (2)	17 (1)	T:0.002, TxO: 0.005 (O↑+T, T↑+O)	11 (3)	15 (3)	15 (5)	10 (3)	
**Valencene**	0 (0)	0 (0)	0 (0)	0 (0)		3 (2)	0 (0)	3 (2)	0 (0)	
[1]***unknown oST 1***	0 (0)	0 (0)	0 (0)	1 (1)		3 (2)	4 (4)	3 (2)	9 (3)	T:0.036↑
[2]***unknown oST 2***	143 (26)	273 (54)	137 (35)	341 (20)	T:0.004 ↑	457 (78)	388 (62)	499 (105)	399 (36)	
Caryophyllene oxide	0 (0)	0 (0)	0 (0)	0 (0)		1 (1)	2 (2)	0 (0)	2 (2)	

[1] potentially nerolidol or farnesol, [2]potentially endo-1-bourbananol or 1,6-germacradien-5-ol

Supplementary Table 4. Terpene concentrations (µg/g FW) of current and previous year needles of Norway spruce seedlings exposed to elevated ozone and elevated temperature alone or in combination on week 26 2010. Compounds are listed in order of their retention time. Non-oxygenated monoterpenes in normal font, *oxygenated monoterpenes* in *cursive*, **non-oxygenated sesquiterpenes (nST) emboldened**, ***oxygenated sesuiterpenes (oST) emboldened, cursive***. AOAT = ambient ozone, ambient temperature, AOET = ambient ozone, elevate temperature, EOAT = elevated ozone, ambient temperature, EOET = elevated ozone, elevated temperature. Values are means (SE) of four treatment replicates. P-values < 0.05 for the main effects of warming (T) and ozone (O) and P-values <0.1 for the TxO interaction from Mixed Models ANOVA are shown. Significant (*P*<0.05, Bonferroni test) simple main effects of interactions explained in parentheses (e.g., T↓+O = warming reduces under elevated ozone, O↑-T = elevated ozone increases in ambient temperature). Increasing ↑ and decreasing ↓ main effects shown. Tr: *P*-values from Kruskal-Wallis test (differences between treatments, when *P*<0.05 for Bonferroni test).

	Current year needles					Previous year needles				
Compound	AOAT	AOET	EOAT	EOET	P	AOAT	AOET	EOAT	EOET	P
Tricyclene	63 (1)	52 (6)	60 (11)	53 (8)		100 (11)	101 (5)	101 (12)	95 (14)	
α-Pinene	369 (37)	297 (39)	363 (58)	316 (32)		633 (72)	579 (51)	636 (89)	599 (47)	
Camphene	618 (12)	516 (43)	598 (91)	518 (67)		949 (87)	963 (49)	1008 (151)	878 (99)	
Sabinene	30 (3)	57 (5)	43 (15)	66 (11)	T:0.038↑	63 (7)	74 (8)	68 (15)	113 (19)	
β-Pinene	65 (11)	63 (11)	78 (30)	45 (2)		145 (27)	102 (29)	133 (46)	124 (20)	
Myrcene	218 (18)	170 (18)	232 (83)	224 (32)		281 (25)	217 (23)	275 (72)	300 (43)	TxO: 0.047
α-Phellandrene	8 (1)	12 (1)	11 (5)	10 (1)	T:0.015 ↑	6 (1)	10 (3)	8 (3)	8 (3)	
3-Carene	3 (1)	8 (2)	4 (1)	2 (1)	TxO: 0.022 (O↓+T, T↑-O)	33 (6)	41 (9)	14 (5)	9 (6)	O:0.002↓
Limonene	300 (22)	325 (56)	341 (44)	297 (52)		568 (26)	496 (30)	554 (71)	476 (57)	
1,8-Cineole	104 (16)	189 (14)	148 (53)	230 (38)	T:0.046 ↑	224 (34)	264 (41)	237 (64)	384 (60)	
(*E*)-β-Ocimene	2 (2)	20 (4)	8 84)	16 (7)	T:0.012↑	1 (1)	9 (5)	0 (0)	4 (2)	T:0.031↑
(E)-β-Sabinene hydrate	2 (1)	8 (1)	5 (4)	8 (4)		9 (1)	10 (2)	19 (10)	16 (1)	
Terpinolene	27 (1)	21 (1)	29 (8)	20 (3)		17 (2)	16 (2)	15 (3)	10 (3)	
Linalool	8 (1)	14 (1)	13 (4)	13 (3)		16 (1)	20 (2)	21 (4)	23 (2)	T:0.019↑
Camphor	30 (12)	42 (27)	123 (71)	59 (15)		47 (16)	44 (14)	148 (71)	72 (23)	
Exomethyl camphenilol	43 (5)	55 (13)	53 (16)	50 (15)		77 (11)	64 (6)	72 (21)	88 (12)	
Borneol	65 (10)	65 (12)	94 (229	54 (27)		91 (12)	75 (20)	148 (83)	69 (33)	
α-Terpineol	42 (6)	84 (5)	63 (22)	95 (20)	T:0.045↑	75 (9)	102 (13)	82 (18)	133 (23)	T:0.035 ↑
cis-Piperitol	0 (0)	17 (17)	0 (0)	0 (0)		0 (0)	17 (17)	0 (0)	0 (0)	
Berbenone	1 (1)	0 (0)	2 (2)	2 (2)		2 (2)	0 (0)	3 (2)	2 (2)	
α-Fenchylacetate	2 (1)	0 (0)	1 (1)	4 (3)		7 (3)	4 (2)	4 (2)	6 (<0.5)	
Citronellol	8 (5)	15 (5)	8 (4)	19 (11)		27 (12)	25 (7)	26 (13)	25 (10)	
Piperitone	5 (3)	14 (7)	15 (12)	19 (13)		8 (5)	12 (12)	22 (16)	21 (16)	
Bornylacetate	779 (47)	649 (115)	698 (88)	655 (91)		1176 (182)	1223 (67)	1265 (233)	1059 (121)	
Exo-2-hydroxy cineoleacetate	11 (4)	11 (1)	8 (4)	22 (6)		11 (2)	13 (3)	9 (3)	28 (6)	T:0.017, TxO:0.040 (O↑+T, T↑+O
unknown ox-MT	9 (3)	17 (11)	19 (7)	19 (5)		29 (2)	30 (11)	29 (9)	46 (10)	

Supplementary table 4 continues

	Current year needles					Previous year needles				
Compound	AOAT	AOET	EOAT	EOET	P	AOAT	AOET	EOAT	EOET	P
**Longipinene**	2 (1)	1 (1)	2 (1)	2 (2)		6 (3)	4 (2)	5 (1)	9 (5)	
Geranylacetate	2 (2)	4 (2)	4 (2)	3 (1)		15 (2)	14 (1)	20 (7)	2 (1)	
**β-Elemene**	15 (2)	13 (3)	13 (2)	15 (3)		22 (1)	16 (2)	16 (2)	23 (9)	
**Longifolene**	4 (1)	7 (1)	7 (2)	6 (1)		7 (1)	13 (3)	10 (2)	10 (2)	
**β-Caryophyllene**	16 (2)	18 (4)	24 (6)	15 (5)		19 (4)	22 (6)	22 (6)	18 (6)	
**unknown nST**	0 (0)	0 (0)	0 (0)	0 (0)		1 (1)	2 (2)	1 (1)	1 (1)	
**(*E*)-β-farnesene**	4 (<0.5)	4 (<0.5)	4 (<0.5)	4 (1)		5 (<0.5)	5 (1)	4 (<0.5)	5 (1)	
**β-Cubebene**	0 (0)	0 (0)	0 (0)	2 (2)		4 (4)	0 (0)	3 (2)	2 (2)	
**α-Humulene**	12 (2)	13 (3)	17 (5)	10 (4)		13 (3)	15 (5)	15 (5)	13 (5)	
**α-Amorphene**	0 (0)	0 (0)	0 (0)	0 (0)		7 (3)	9 (2)	4 (2)	8 (3)	
**Germacrene-D**	20 (1)	21 (3)	24 (6)	21 (2)		34 (3)	26 (6)	28 (5)	25 (6)	
**α-Muurolene**	23 (5)	19 (2)	15 (4)	29 (3)	TxO:0.048 (T↑+O)	21 (4)	15 (3)	7 (4)	19 (10)	TxO:0.037 (T↑+O)
**γ-Cadinene**	16 (1)	14 (3)	12 (1)	16 (3)		28 (3)	18 (2)	24 (4)	35 (15)	
**δ-Cadinene**	13 (1)	11 (3)	10 (1)	14 (3)		26 (5)	19 (3)	24 (4)	35 (14)	
**Valencene**	2 (1)	1 (1)	1 (1)	1 (1)		1 (1)	3 (3)	3 (3)	8 (4)	
[1]unknown oST 1	2 (2)	4 (2)	1 (1)	3 (3)		11 (4)	14 (7)	9 (4)	12 (7)	
[2]unknown oST 2	468 (94)	389 (17)	446 (44)	534 (121)		532 (48)	380 (43)	393 (57)	542 (227)	
Caryophyllene oxide	0 (0)	0 (0)	0 (0)	0 (0)		0 (0)	0 (0)	1 (1)	2 (2)	

[1] potentially nerolidol or farnesol, [2]potentially endo-1-bourbananol or 1,6-germacradien-5-ol

Supplementary Table 5. Terpene concentrations (µg/g FW) of current and previous year needles of Norway spruce seedlings exposed to elevated ozone and elevated temperature alone or in combination on week 31 2010. Compounds are listed in order of their retention time. Non-oxygenated monoterpenes in normal font, *oxygenated monoterpenes* in *cursive*, **non-oxygenated sesquiterpenes (nST) emboldened**, ***oxygenated sesuiterpenes (oST) emboldened, cursive***. AOAT = ambient ozone, ambient temperature, AOET = ambient ozone, elevate temperature, EOAT = elevated ozone, ambient temperature, EOET = elevated ozone, elevated temperature. Values are means (SE) of four treatment replicates. P-values < 0.05 for the main effects of warming (T) and ozone (O) and P-values <0.1 for the TxO interaction from Mixed Models ANOVA are shown. Significant (*P*<0.05, Bonferroni test) simple main effects of interactions explained in parentheses (e.g., T↓+O = warming reduces under elevated ozone, O↑-T = elevated ozone increases in ambient temperature). Increasing ↑ and decreasing ↓ main effects shown. Tr: *P*-values from Kruskal-Wallis test (differences between treatments, when *P*<0.05 for Bonferroni test).

	Current year needles					Previous year needles				
Compound	AOAT	AOET	EOAT	EOET	P	AOAT	AOET	EOAT	EOET	P
Tricyclene	103 (6)	85 (3)	85 (11)	80 (11)	T:0.041↓	127 (13)	110 (2)	109 (12)	113 (18)	
α-Pinene	652 (75)	451 (16)	586 (83)	502 (60)	T: 0.021↓	790 (90)	663 (38)	748 (70)	774 (107)	
Camphene	1060 (81)	860 (27)	862 (113)	830 (91)		1251 (128)	1110 (20)	1112 (105)	1182 (159)	
Sabinene	70 (1)	54 (8)	60 (12)	38 (3)	T:0.037↓	79 (8)	69 (10)	67 (7)	61 (7)	
β-Pinene	90 (9)	57 (2)	75 (14)	63 (8)	T:0.028 ↓	134 (20)	107 (17)	105 (12)	105 (12)	
Myrcene	217 (20)	159 (20)	180 (30)	175 (16)	T:0.028 ↓	245 (24)	210 (32)	207 (17)	277 (16)	TxO:0.003 (T↑+O)
α-Phellandrene	11 (1)	7 (1)	8 (1)	8 (2)	T:0.005, OxT:0.007 (T↓-O)	9 (1)	5(1)	7 (1)	7 (1)	T:<0.001, TxO: <0.001 (T↓-O)
3-Carene	9 (3)	8 (2)	12 (4)	18 (10)		20 (6)	14 (4)	31 (12)	16 (5)	
Limonene	518 (51)	420 (17)	384 (43)	471 (44)	TxO: 0.057 (O↓-T)	592 (43)	571 (23)	475 (46)	748 (32)	T:0.007, TxO: 0.004 (T↑+O, O ↓-T, O↑+T)
1,8-Cineole	309 (12)	250 (41)	252 (46)	150 (24)	T:0.030 ↓	355 (37)	309 (49)	275 (31)	261 (41)	
(*E*)-β-Ocimene	7 (3)	5 (5)	16 (5)	13 (5)		6 (2)	0 (0)	11 (4)	3 (2)	T:0.009 ↓
(E)-β-Sabinene hydrate	14 (1)	6 (3)	9 (3)	3 (2)	T:0.015 ↓	16 (3)	8 (3)	12 (2)	10 (2)	
Terpinolene	23 (2)	14 (1)	22 (3)	16 (2)	T:0.003↓	20 (4)	8 (<0.5)	16 (2)	9 (1)	T:0.001↓
Linalool	17 (<0.5)	13 (1)	15 (3)	11 (1)	T:0.003↓	21 (2)	19 (2)	18 (1)	20 (1)	
Camphor	53 (13)	64 (28)	66 (8)	44 (23)		43 (11)	81 (33)	85 (23)	76 (35)	
Exomethyl camphenilol	118 (10)	98 (11)	72 (10)	68 (28)		143 (19)	198 (19)	101 (14)	104 (38)	
Borneol	183 (77)	48 (10)	89 (28)	90 (42)		163 (64)	69 (8)	121 (47)	158 (58)	
α-Terpineol	103 (6)	72 (9)	81 (18)	50 (10)	T:0.021↓	124 (16)	97 (13)	89 (12)	94 (15)	
cis-Piperitol	3 (3)	0 (0)	0 (0)	3 (3)		4 (4)	0 (0)	4 (4)	0 (0)	
Berbenone	2 (2)	0 (0)	4 (2)	1 (1)		3 (1)	1 (1)	4 (2)	4 (1)	
α-Fenchylacetate	3 (2)	6 (1)	2 (1)	3 (2)		8 (2)	11 (2)	5 (3)	3 (3)	O:0.025 ↓

Supplementary Table 5 continues

	Current year needles					Previous year needles				
Compound	AOAT	AOET	EOAT	EOET	P	AOAT	AOET	EOAT	EOET	P
Citronellol	34 (7)	14 (3)	34 (31)	23 (9)		50 (12)	29 (4)	43 (35)	38 (10)	
Piperitone	16 (9)	9 (5)	9 (9)	5 (5)		21 (13)	11 (7)	14 (12)	10 (8)	
Bornylacetate	1450 (176)	1251 (55)	1214 (192)	1287 (189)		1737 (177)	1501 (56)	1553 (130)	1722 (304)	
Exo-2-hydroxy cineoleacetate	13 (5)	13 (5)	7 (3)	12 (9)		23 (8)	21 (4)	13 (5)	12 (7)	
unknown ox-MT	30 (7)	35 (8)	21 (8)	22 (11)		40 (8)	47 (8)	31 (12)	33 (17)	
**Longipinene**	5 (2)	2 (1)	3 (3)	2 (1)		7 (1)	7 (4)	5 (4)	5 (3)	
Geranylacetate	14 (4)	10 (6)	8 (6)	20 (5)	TxO:0.065 (T↑+O)	21 (4)	10 (5)	13 (6)	19 (4)	TxO:0.046
**β-Elemene**	19 (3)	11 (3)	21 (5)	15 (3)		21 (1)	14 (4)	22 (3)	19 (3)	
**Longifolene**	10 (1)	8 (2)	10 (4)	9 (2)		12 (1)	15 (6)	13 (5)	13 (2)	
**β-Caryophyllene**	36 (12)	26 (6)	35 (15)	35 (18)		38 (14)	27 (8)	34 (12)	38 (20)	
**unknown nST**	3 (3)	1 (1)	1 (1)	1 (1)		3 (3)	1 (1)	1 (1)	2 (2)	
**(*E*)-β-farnesene**	4 (<0.5)	3 (<0.5)	4 (1)	4 (<0.5)		5 (<0.5)	5 (<0.5)	5 (1)	5 (<0.5)	
**β-Cubebene**	2 (2)	0 (0)	0 (0)	0 (0)		1 (1)	0 (0)	0 (0)	0 (0)	
**α-Humulene**	26 (9)	18 (4)	26 (11)	24 (12)		29 (11)	20 (6)	26 (10)	26 (14)	
**α-Amorphene**	2 (2)	2 (2)	0 (0)	1 (1)		5 (2)	9 (4)	8 (5)	8 (1)	
**Germacrene-D**	50 (14)	36 (6)	61 (15)	37 (8)		58 (12)	54 (12)	52 (16)	31 (8)	
**α-Muurolene**	18 (8)	10 (6)	23 (5)	17 (6)		15 (4)	5 (3)	24 (4)	2 (2)	T:<0.001 TxO:0.094 T↓-O, T↓+O)
**γ-Cadinene**	30 (2)	17 (5)	29 (6)	26 (5)		27 (2)	16 (5)	33 (2)	23 (4)	T:0.005↓
**δ-Cadinene**	25 (2)	14 (4)	24 (6)	23 (4)		24 (2)	14 (4)	27 (2)	19 (2)	T:0.006↓
**Valencene**	3 (2)	0 (0)	2 (2)	2 (2)		1 (1)	2 (2)	2 (2)	0 (0)	
[1]unknown oST 1	6 (4)	5 (3)	11 (4)	6 (3)		9 (4)	10 (4)	10 (4)	18 (4)	
[2]unknown oST 2	549 (47)	339 (51)	607 (130)	455 (27)	T:0.042↓	466 (33)	240 (60)	534 (81)	329 (48)	T:0.008↓
Caryophyllene oxide	0 (0)	0 (0)	0 (0)	0 (0)		2 (1)	0 (0)	1 (1)	7 (7)	

[1] potentially nerolidol or farnesol, [2]potentially endo-1-bourbananol or 1,6-germacradien-5-ol

Supplementary Table 6. Terpene concentrations (µg/g FW) of current and previous year needles of Norway spruce seedlings exposed to elevated ozone and elevated temperature alone or in combination on week 36 2010. Compounds are listed in order of their retention time. Non-oxygenated monoterpenes in normal font, *oxygenated monoterpenes* in *cursive*, **non-oxygenated sesquiterpenes (nST) emboldened**, ***oxygenated sesuiterpenes (oST) emboldened, cursive***. AOAT = ambient ozone, ambient temperature, AOET = ambient ozone, elevate temperature, EOAT = elevated ozone, ambient temperature, EOET = elevated ozone, elevated temperature. Values are means (SE) of four treatment replicates. P-values < 0.05 for the main effects of warming (T) and ozone (O) and P-values <0.1 for the TxO interaction from Mixed Models ANOVA are shown. Significant (*P*<0.05, Bonferroni test) simple main effects of interactions explained in parentheses (e.g., T↓+O = warming reduces under elevated ozone, O↑-T = elevated ozone increases in ambient temperature). Increasing ↑ and decreasing ↓ main effects shown. Tr: *P*-values from Kruskal-Wallis test (differences between treatments, when *P*<0.05 for Bonferroni test).

	Current year needles					Previous year needles				
Compound	AOAT	AOET	EOAT	EOET	P	AOAT	AOET	EOAT	EOET	P
Tricyclene	69 (2)	67 (8)	82 (8)	48 (4)	T:0.019, TxO:0.029 (T↓+O)	86 (13)	111 (12)	121 (13)	96 (7)	TxO:0.065
α-Pinene	442 (40)	411 (75)	490 (85)	328 (38)		593 (94)	718 (53)	744 (134)	657 (55)	
Camphene	698 (44)	649 (61)	784 (54)	527 (50)	T:0.022, TxO:0.085 (T↓+O)	912 (91)	1122 (95)	1188 (102)	1029 (67)	TxO:0.077 (O↑-T)
Sabinene	68 (9)	51 (9)	78 (15)	48 (15)		74 (18)	80 (7)	75 (18)	89 (22)	
β-Pinene	96 (14)	62 (2)	94 (6)	62 (8)	T:0.017↓	101 (23)	101 (12)	134 (16)	103 (17)	
Myrcene	250 (39)	192 (29)	246 (50)	208 (87)		239 (32)	258 (24)	253 (54)	326 (99)	
α-Phellandrene	9 (3)	5 (1)	11 (1)	5 (1)	T:0.011↓	6 (1)	3 (<0.5)	7 (1)	5 (1)	T:0.001↓
3-Carene	7 (2)	14 (6)	11 (4)	9 (4)		10 (4)	29 (14)	25 (8)	15 (7)	
Limonene	411 (34)	304 (61)	373 (47)	269 (27)	T:0.042↓	592 (69)	589 (136)	632 (87)	577 (51)	
1,8-Cineole	228 (34)	172 (36)	266 (41)	162 (43)		286 (58)	343 (48)	350 (64)	412 (100)	
(*E*)-β-Ocimene	5 (3)	10 (5)	9 (2)	3 (3)		4 (4)	1 (1)	8 (5)	2 (2)	
(E)-β-Sabinene hydrate	5 (2)	6 (3)	9 (3)	5 (3)		11 (2)	9 (<0.5)	15 (6)	17 (4)	
Terpinolene	21 (3)	14 (2)	24 (4)	14 (4)	T:0.011↓	12 (3)	6 (2)	13 (4)	8 (2)	
Linalool	14 (2)	10 (3)	15 (3)	10 (3)		17 (3)	19 (2)	19 (4)	24 (6)	
Camphor	71 (27)	17 (2)	139 (32)	113 (66)		51 (19)	46 (11)	147 (51)	181 (100)	
Exomethyl camphenilol	52 (13)	54 (13)	85 (20)	35 (2)	TxO:0.094 (T↓+O)	79 (25)	94 (11)	128 (27)	86 (9)	
Borneol	148 (65)	49 (5)	156 (45)	79 (17)		111 (67)	113 (4)	137 (25)	129 (30)	
α-Terpineol	96 (20)	61 (15)	93 (18)	59 (15)		94 (25)	116 (13)	106 (26)	146 (33)	
cis-Piperitol	0 (0)	0 (0)	0 (0)	0 (0)		3 (3)	0 (0)	0 (0)	0 (0)	
Berbenone	0 (0)	0 (0)	2 (2)	0 (0)		0 (0)	1 (1)	2 (2)	3 (3)	
α-Fenchylacetate	2 (1)	1 (1)	1 (1)	0 (0)		6 (4)	3 (3)	6 (4)	3 (2)	
Citronellol	5 (4)	3 (3)	3 (1)	3 (2)		28 (8)	12 (3)	21 (6)	36 (14)	TxO:0.092
Piperitone	9 (8)	2 (2)	18 (10)	5 (2)		15 (11)	4 (4)	34 (19)	12 (5)	
Bornylacetate	843 (50)	925 (112)	806 (90)	668 (48)		1361 (66)	1527 (138)	1414 (163)	1349 (122)	
Exo-2-hydroxy cineoleacetate	16 (8)	4 (3)	6 (3)	1 (1)		26 (8)	12 (8)	14 (8)	16 (7)	

Supplementary Table 6 continues

	Current year needles					Previous year needles				
Compound	AOAT	AOET	EOAT	EOET	P	AOAT	AOET	EOAT	EOET	P
unknown ox-MT	30 (11)	12 (7)	19 (4)	6 (5)		48 (12)	23 (14)	38 (8)	24 (9)	T:0.050↓
**Longipinene**	3 (2)	0 (0)	6 (4)	2 (2)		8 (4)	1 (1)	8 (4)	6 (6)	
Geranylacetate	1 (1)	5 (3)	2 (2)	0 (0)		14 (4)	20 (15)	17 (4)	4 (3)	
**β-Elemene**	25 (13)	9 (1)	16 (1)	6 (2)		22 (1)	11 (4)	23 (3)	15 (5)	T:0.011↓
**Longifolene**	7 (1)	8 (5)	9 (2)	3 (1)		11 (2)	13 (9)	13 (3)	6 (3)	
**β-Caryophyllene**	25 (7)	33 (10)	26 (9)	16 (9)		26 (4)	24 (5)	26 (7)	23 (7)	
**unknown nST**	0 (0)	0 (0)	0 (0)	0 (0)		0 (0)	0 (0)	1 (1)	0 (0)	
**(*E*)-β-farnesene**	3 (1)	3 (1)	5 (1)	2 (<0.5)	TxO: 0.041 (T↓+O)	5 (1)	5 (2)	5 (1)	4 (1)	
**β-Cubebene**	0 (0)	0 (0)	2 (1)	0 (0)		0 (0)	0 (0)	0 (0)	0 (0)	
**α-Humulene**	19 (6)	25 (8)	18 (8)	12 (6)		18 (2)	18 (4)	18 (7)	17 (6)	
**α-Amorphene**	6 (5)	0 (0)	6 (3)	0 (0)		5 (2)	4 (2)	5 (4)	5 (3)	
**Germacrene-D**	21 (7)	19 (2)	21 (1)	15 (9)		36 (8)	15 (2)	43 (9)	36 (3)	
**α-Muurolene**	33 (17)	10 (5)	22 (4)	8 (3)		16 (1)	1 (1)	4 (2)	8 (5)	TxO:0.013 (T↓-O, O↓-T)
**γ-Cadinene**	40 (17)	16 (1)	27 (4)	14 (2)		34 (1)	15 (9)	25 (5)	21 (7)	T:0.031↓
**δ-Cadinene**	36 (14)	17 (2)	28 (5)	16 (4)		30 (2)	12 (6)	21 (4)	18 (6)	T:0.025, TxO: 0.081 (T↓-O)
**Valencene**	12 (5)	3 (2)	12 (2)	3 (2)	T:0.013 ↓	0 (0)	0 (0)	0 (0)	0 (0)	
[1]unknown oST 1	1 (1)	4 (2)	1 (1)	0 (0)		12 (4)	18 (7)	5 (5)	10 (3)	
[2]unknown oST 2	406 (55)	253 (15)	417 (36)	231 (38)	T:0.003↓	524 (51)	148 (64)	463 (73)	311 (122)	T:0.013↓
Caryophyllene oxide	0 (0)	2 (2)	1 (1)	0 (0)		2 (2)	11 (2)	4 (3)	5 (5)	

[1] potentially nerolidol or farnesol, [2]potentially endo-1-bourbananol or 1,6-germacradien-5-ol

Supplementary Table 7. Actual (non-standardized) and standardized (+30 °C, isoprene also to PAR-level 1000 µmol m^{-2} s^{-1}) emission rates (ng/g-1 DW h-1) of Norway spruce seedlings exposed to elevated ozone and elevated temperature alone or in combination on week 22 in 2010. Compounds are listed in order of their retention time. Isoprene and non-oxygenated monoterpenes in normal font, *oxygenated monoterpenes* in *cursive*, **non-oxygenated sesquiterpenes (nST) emboldened**, ***GLVs and methylsalicylate emboldened, cursive***. AOAT = ambient ozone, ambient temperature, AOET = ambient ozone, elevate temperature, EOAT = elevated ozone, ambient temperature, EOET = elevated ozone, elevated temperature. Values are means (SE) of four treatment replicates. P-values < 0.05 for the main effects of warming (T) and ozone (O) and P-values <0.1 for the TxO interaction from Mixed Models ANOVA are shown. Significant (*P*<0.05, Bonferroni test) simple main effects of interactions explained in parentheses (e.g., T↓+O = warming reduces under elevated ozone, O↑-T = elevated ozone increases in ambient temperature). Increasing ↑ and decreasing ↓ main effects shown when interaction not found. Tr: *P*-values from Kruskal-Wallis test (differences between treatments, when *P*<0.05 for Bonferroni test).

	Non-standardized					Standardized				
Compound	AOAT	AOET	EOAT	EOET	*P*	AOAT	AOET	EOAT	EOET	*P*
Isoprene	26 (16)	13 (10)	37 (25)	2 (1)		43 (24)	60 (50)	124 (105)	10 (6)	
Tricyclene	21 (4)	15 (2)	24 (2)	14 (4)	T:0.040 ↓	41 (11)	26 (6)	48 (10)	30 (11)	T:0.047 ↓
α-Pinene	445 (126)	440 (133)	385 (47)	347 (140)		918 (349)	813 (213)	758 (153)	733 (375)	
Camphene	186 (25)	112 (22)	197 (26)	123 (34)	T:0.022 ↓	362 (81)	187 (40)	376 (67)	254 (102)	T:0.055 ↓
Sabinene	28 (19)	17 (10)	10 (8)	16 (9)		56 (40)	34 (26)	25 (21)	33 (23)	
β-Pinene	318 (128)	344 (93)	200 (30)	217 (125)		674 (305)	552 (134)	401 (92)	485 (310)	
Myrcene	233 (96)	122 (16)	181 (35)	100 (60)		417 (166)	205 (36)	346 (73)	218 (149)	
(Z)-Hexenyl-acetate	0 (0)	0 (0)	0 (0)	0 (0)		0 (0)	0 (0)	0 (0)	0 (0)	
α-Phellandrene	9 (4)	8 (1)	6 (1)	6 (4)		17 (8)	14 (3)	13 (4)	13 (9)	
α-Terpinene	4 (2)	4 (1)	2 (1)	3 (1)		8 (5)	6 (2)	5 (2)	6 (3)	
3-Carene	348 (271)	256 (155)	195 (104)	165 (119)		696 (576)	516 (387)	465 (291)	350 (294)	
Cymene	9 (3)	10 (1)	8 (2)	9 (3)		17 (7)	17 (4)	18 (6)	18 (9)	
Limonene	1271 (346)	501 (133)	778 (43)	440 (308)	T: 0.027 ↓	2215 (421)	785 (158)	1486 (198)	969 (757)	T: 0.026 ↓
β-Phellandrene	148 (78)	173 (64)	92 (26)	165 (127)		314 (170)	280 (102)	184 (50)	374 (307)	
1,8-Cineole	18 (7)	3 (1)	8 (3)	10 (2)	T: 0.040 TxO: 0.014 (T↓ -O)	33 (13)	6 (2)	13 (3)	18 (2)	T:0.035, TxO: 0.015 (T↓-O)
γ-Terpinene	6 (4)	5 (2)	3 (1)	4 (2)		13 (9)	9 (4)	6 (2)	8 (4)	
Terpinolene	32 (21)	15 (5)	9 (3)	12 (8)		63 (48)	27 (13)	19 (9)	25 (20)	
Linalool	0 (0)	0 (0)	0 (0)	1 (1)	Tr:0.048	0 (0)	0 (0)	0 (0)	2 (2)	Tr:0.048
Camphor	3 (1)	2 (<0.5)	1 (<0.5)	3 (1)	TxO: 0.015 (T↓-O, O↑+T)	6 (2)	4 (1)	2 (<0.5)	5 (2)	TxO: 0.016 (T↓-O , O↑+T)
Borneol	3 (<0.5)	2 (<0.5)	2 (<0.5)	3 (1)	TxO: 0.025 (T↓-O, O↓-T)	6 (1)	3 (<0.5)	3 (1)	5 (1)	TxO: 0.037 (T↓-O, O↓-T)
Pinocarvone	<0.5 (<0.5)	<0.5 (<0.5)	1 (1)	<0.5 (<0.5)		1 (1)	1 (<0.5)	1 (1)	1 (1)	
Isopinocamphone	<0.5 (<0.5)	0 (0)	0 (0)	1 (1)		<0.5 (<0.5)	0 (0)	0 (0)	2 (2)	
Terpinen-4-ol	0 (0)	1 (1)	0 (0)	1 (1)		0 (0)	1 (1)	0 (0)	1 (1)	

Supplementary Table 7 continues

	Non-standardized					Standardized				
Compound	AOAT	AOET	EOAT	EOET	*P*	AOAT	AOET	EOAT	EOET	*P*
α-Terpineol	1 (1)	<0.5 (<0.5)	0 (0)	<0.5 (<0.5)		2 (1)	<0.5 (<0.5)	0 (0)	1 (1)	
Methylsalicylate	0 (0)	0 (0)	0 (0)	0 (0)		0 (0)	0 (0)	0 (0)	0 (0)	
Myrtenal	1 (<0.5)	1 (1)	<0.5 (<0.5)	1 (1)		1 (1)	1 (1)	1 (1)	2 (2)	
Berbenone	0 (0)	0 (0)	0 (0)	0 (0)		0 (0)	0 (0)	0 (0)	0 (0)	
Piperitone	0 (0)	0 (0)	1 (1)	0 (0)		0 (0)	0 (0)	1 (1)	0 (0)	
Bornylacetate	64 (16)	23 (9)	45 (13)	30 (12)	T:0.029 ↓	122 (37)	38 (14)	83 (20)	63 (32)	T: 0.037 ↓
**α-Cubebene**	0 (0)	1 (1)	0 (0)	0 (0)		0 (0)	2 (2)	0 (0)	0 (0)	
**Longipinene**	6 (2)	5 (1)	5 (1)	4 (2)		22 (10)	14 (7)	17 (6)	17 (10)	
**α-Ylangene**	1 (<0.5)	2 (2)	<0.5 (<0.5)	1 (1)		2 (1)	4 (4)	2 (2)	2 (2)	
**Copaene**	0 (0)	0 (0)	0 (0)	0 (0)		0 (0)	0 (0)	0 (0)	0 (0)	
**Longicyclene**	1 (<0.5)	1 (<0.5)	<0.5 (<0.5)	1 (1)	T:0.041 ↓	2 (1)	3 (2)	2 (1)	6 (4)	T: 0.063
**β-Bourbobene**	2 (1)	16 (14)	<0.5 (<0.5)	5 (4)		5 (2)	38 (33)	2 (2)	19 (19)	
**unknown nST 1**	0 (0)	0 (0)	0 (0)	0 (0)		0 (0)	0 (0)	0 (0)	0 (0)	
**unknown nST 2**	0 (0)	0 (0)	0 (0)	0 (0)		0 (0)	0 (0)	0 (0)	0 (0)	
**Isoledene**	<0.5 (<0.5)	4 (4)	0 (0)	1 (1)		1 (1)	9 (9)	0 (0)	4 (3)	
**unknown nST 3**	0 (0)	0 (0)	0 (0)	0 (0)		0 (0)	0 (0)	0 (0)	0 (0)	
**unknown nST 4**	0 (0)	0 (0)	0 (0)	0 (0)		0 (0)	0 (0)	0 (0)	0 (0)	
**Longifolene**	5 (1)	7 (2)	5 (<0.5)	8 (4)		10 (3)	13 (4)	9 (1)	18 (11)	
**unknown nST 5**	0 (0)	0 (0)	0 (0)	0 (0)		0 (0)	0 (0)	0 (0)	0 (0)	
**β-Caryophyllene**	1 (<0.5)	2 (1)	1 (1)	1 (<0.5)		4 (2)	4 (3)	3 (2)	4 (2)	
**(*E*)-β-farnesene**	<0.5 (<0.5)	0 (0)	0 (0)	0 (0)		1 (1)	0 (0)	0 (0)	0 (0)	
**β-Cubebene**	0 (0)	0 (0)	0 (0)	0 (0)		0 (0)	0 (0)	0 (0)	0 (0)	
**unknown nST 6**	0 (0)	0 (0)	0 (0)	0 (0)		0 (0)	0 (0)	0 (0)	0 (0)	
**unknown nST 7**	0 (0)	0 (0)	0 (0)	0 (0)		0 (0)	0 (0)	0 (0)	0 (0)	
**α-Humulene**	1 (<0.5)	1 (1)	1 (<0.5)	<0.5 (<0.5)		1 (1)	2 (2)	1 (<0.5)	1 (<0.5)	
**unknown nST 8**	0 (0)	0 (0)	0 (0)	0 (0)		0 (0)	0 (0)	0 (0)	0 (0)	
**(*E,E*)-α-Farnesene**	0 (0)	0 (0)	0 (0)	1 (1)		0 (0)	0 (0)	0 (0)	3 (3)	
**Germacrene-D**	0 (0)	2 (2)	0 (0)	<0.5 (<0.5)		0 (0)	4 (4)	0 (0)	2 (2)	
**unknown nST 8a**	0 (0)	0 (0)	0 (0)	0 (0)		0 (0)	0 (0)	0 (0)	0 (0)	
**unknown nST 9**	0 (0)	0 (0)	0 (0)	0 (0)		0 (0)	0 (0)	0 (0)	0 (0)	
**unknown nST 10**	0 (0)	1 (1)	0 (0)	0 (0)		0 (0)	3 (3)	0 (0)	0 (0)	
**γ-Cadinene**	0 (0)	1 (1)	0 (0)	0 (0)		0 (0)	2 (2)	0 (0)	0 (0)	
**δ-Cadinene**	<0.5 (<0.5)	2 (1)	<0.5 (<0.5)	1 (1)	T:0.023 ↑	1 (<0.5)	3 (2)	<0.5 (<0.5)	2 (1)	T:0.022↑
**Bisabolene**	0 (0)	0 (0)	0 (0)	0 (0)		0 (0)	0 (0)	0 (0)	0 (0)	

Supplementary Table 8. Actual (non-standardized) and standardized (+30 °C, isoprene also to PAR-level 1000 µmol m^{-2} s^{-1}) emission rates (ng/g-1 DW h-1) of Norway spruce seedlings exposed to elevated ozone and elevated temperature alone or in combination on week 26 in 2010. Compounds are listed in order of their retention time. Isoprene and non-oxygenated monoterpenes in normal font, *oxygenated monoterpenes* in *cursive*, **non-oxygenated sesquiterpenes (nST) emboldened**, ***GLVs and methylsalicylate emboldened, cursive***. AOAT = ambient ozone, ambient temperature, AOET = ambient ozone, elevate temperature, EOAT = elevated ozone, ambient temperature, EOET = elevated ozone, elevated temperature. Values are means (SE) of four treatment replicates. P-values < 0.05 for the main effects of warming (T) and ozone (O) and P-values <0.1 for the TxO interaction from Mixed Models ANOVA are shown. Significant (*P*<0.05, Bonferroni test) simple main effects of interactions explained in parentheses (e.g., T↓+O = warming reduces under elevated ozone, O↑-T = elevated ozone increases in ambient temperature). Increasing ↑ and decreasing ↓ main effects shown when interaction not found. Tr: *P*-values from Kruskal-Wallis test (differences between treatments, when *P*<0.05 for Bonferroni test).

	Non-standardized					Standardized				
Compound	AOAT	AOET	EOAT	EOET	*P*	AOAT	AOET	EOAT	EOET	*P*
Isoprene	15 (11)	<0.5 (<0.5)	8 (3)	9 (9)		23 (19)	1 (<0.5)	19 (7)	14 (12)	
Tricyclene	12 (2)	7 (3)	13 (3)	9 (2)		13 (3)	11 (6)	30 (10)	16 (8)	
α-Pinene	511 (196)	209 (129)	300 (129)	319 (163)		586 (263)	374 (311)	560 (173)	641 (445)	
Camphene	91 (18)	60 (25)	130 (9)	73 (17)	T:0.028 ↓	98 (24)	93 (62)	286 (67)	132 (61)	O:0.080 ↑
Sabinene	20 (8)	7 (3)	8 (3)	10 (4)		23 (10)	10 (5)	14 (4)	15 (6)	
β-Pinene	243 (86)	108 (59)	166 (112)	159 (58)		266 (91)	182 (142)	284 (161)	300 (171)	
Myrcene	102 (34)	45 (24)	64 (27)	75 (26)		111 (34)	75 (58)	112 (35)	122 (46)	
(Z)-Hexenyl-acetate	0 (0)	2 (2)	0 (0)	0 (0)		0 (0)	1 (1)	0 (0)	0 (0)	
α-Phellandrene	7 (2)	4 (2)	4 (2)	3 (1)		8 (3)	6 (4)	7 (2)	6 (3)	
α-Terpinene	3 (1)	3 (1)	2 (1)	2 (<0.5)		3 (1)	4 (2)	3 (2)	3 (1)	
3-Carene	177 (80)	47 (28)	24 (17)	59 (55)		201 (105)	49 (19)	45 (29)	71 (64)	
Cymene	8 (2)	7 (2)	5 (2)	5 (<0.5)		10 (3)	10 (5)	10 (3)	9 (2)	
Limonene	542 (383)	314 (184)	196 (65)	190 (73)		643 (487)	553 (397)	395 (113)	364 (216)	
β-Phellandrene	198 (112)	58 (30)	90 (53)	90 (55)		207 (110)	93 (71)	158 (75)	187 (144)	
1,8-Cineole	15 (4)	22 (12)	24 (11)	31 (10)		15 (3)	25 (11)	42 (17)	45 (7)	O:0.068 ↑
γ-Terpinene	3 (1)	2 (1)	2 (1)	2 (<0.5)		3 (1)	3 (2)	3 (1)	3 (<0.5)	
Terpinolene	13 (3)	4 (2)	4 (2)	5 (2)		14 (5)	6 (4)	8 (3)	7 (2)	
Linalool	<0.5 (<0.5)	2 (1)	<0.5 (<0.5)	2 (1)	T: 0.044↑	<0.5 (<0.5)	3 (2)	1 (1)	3 (1)	T: 0.066 ↑
Camphor	5 (1)	4 (1)	12 (7)	5 (2)		5 (2)	6 (3)	21 (10)	8 (3)	
Borneol	1 (<0.5)	1 (<0.5)	1 (1)	<0.5 (<0.5)		1 (<0.5)	1 (1)	3 (2)	<0.5 (<0.5)	
Pinocarvone	1 (1)	0 (0)	0 (0)	0 (0)		1 (1)	0 (0)	0 (0)	0 (0)	
Isopinocamphone	<0.5 (<0.5)	0 (0)	<0.5 (<0.5)	<0.5 (<0.5)		<0.5 (<0.5)	0 (0)	1 (1)	1 (1)	
Terpinen-4-ol	0 (0)	1 (1)	1 (1)	<0.5 (<0.5)		0 (0)	1 (1)	1 (1)	1 1)	
α-Terpineol	1 (<0.5)	4 (2)	1 (1)	2 (<0.5)		1 (<0.5)	5 (3)	2 (2)	3 (<0.5)	T:0.062 ↑
Methylsalicylate	1 (1)	62 (53)	<0.5 (<0.5)	3 (2)		1 (1)	137 (130)	1 (1)	7 (5)	
Myrtenal	1 (1)	1 (1)	0 (0)	<0.5 (<0.5)		1 (1)	1 (1)	0 (0)	1 (1)	
Berbenone	1 (1)	0 (0)	0 (0)	0 (0)		1 (1)	0 (0)	0 (0)	0 (0)	
Piperitone	0 (0)	2 (2)	<0.5 (<0.5)	1 (1)		0 (0)	1 (1)	1 (1)	1 (1)	
Bornylacetate	12 (4)	9 (3)	22 (6)	8 (2)		11 (2)	13 (7)	53 (22)	14 (6)	O:0.093 ↑

Supplementary Table 8 continues

	Non-standardized					Standardized				
Compound	AOAT	AOET	EOAT	EOET	*P*	AOAT	AOET	EOAT	EOET	*P*
**α-Cubebene**	<0.5 (<0.5)	1 (<0.5)	0 (0)	0 (0)		<0.5 (<0.5)	2 (1)	0 (0)	0 (0)	
**Longipinene**	4 (1)	2 (1)	1 (<0.5)	1 (<0.5)		4 (<0.5)	6 (6)	5 (2)	4 (2)	
**α-Ylangene**	1 (<0.5)	1 (<0.5)	0 (0)	<0.5 (<0.5)	O:0.016↓	1 (<0.5)	2 (2)	0 (0)	<0.5 (<0.5)	
**Copaene**	<0.5 (<0.5)	<0.5 (<0.5)	0 (0)	0 (0)		<0.5 (<0.5)	1 (1)	0 (0)	0 (0)	
**Longicyclene**	<0.5 (<0.5)	1 (<0.5)	<0.5 (<0.5)	<0.5 (<0.5)		<0.5 (<0.5)	2 (2)	1 (1)	1 (1)	
**β-Bourbobene**	4 (3)	1 (1)	1 (1)	<0.5 (<0.5)		3 (1)	2 (1)	3 (3)	<0.5 (<0.5)	
**unknown nST 1**	0 (0)	0 (0)	0 (0)	0 (0)		0 (0)	0 (0)	0 (0)	0 (0)	
**unknown nST 2**	0 (0)	0 (0)	0 (0)	0 (0)		0 (0)	0 (0)	0 (0)	0 (0)	
**Isoledene**	1 (<0.5)	2 (1)	<0.5 (<0.5)	0 (0)		1 (<0.5)	4 (3)	1 (1)	0 (0)	
**unknown nST 3**	0 (0)	0 (0)	0 (0)	0 (0)		0 (0)	0 (0)	0 (0)	0 (0)	
**unknown nST 4**	0 (0)	0 (0)	0 (0)	0 (0)		0 (0)	0 (0)	0 (0)	0 (0)	
**Longifolene**	4 (1)	4 (2)	2 (1)	2 (<0.5)		4 (1)	7 (5)	4 (1)	4 (2)	
**unknown nST 5**	<0.5 (<0.5)	0 (0)	0 (0)	0 (0)		<0.5 (<0.5)	0 (0)	0 (0)	0 (0)	
**β-Caryophyllene**	1 (1)	1 (1)	1 (1)	1 (1)		1 (<0.5)	1 (1)	3 (2)	2 (2)	
**(*E*)-β-farnesene**	1 (1)	3 (2)	1 (1)	2 (1)		1 (<0.5)	6 (5)	3 (3)	3 (1)	
**β-Cubebene**	1 (1)	0 (0)	0 (0)	0 (0)		<0.5 (<0.5)	0 (0)	0 (0)	0 (0)	
**unknown nST 6**	0 (0)	0 (0)	0 (0)	0 (0)		0 (0)	0 (0)	0 (0)	0 (0)	
**unknown nST 7**	0 (0)	0 (0)	0 (0)	0 (0)		0 (0)	0 (0)	0 (0)	0 (0)	
**α-Humulene**	1 (<0.5)	<0.5 (<0.5)	1 (<0.5)	0 (0)		1 (<0.5)	<0.5 (<0.5)	1 (1)	0 (0)	
**unknown nST 8**	<0.5 (<0.5)	1 (1)	0 (0)	0 (0)		<0.5 (<0.5)	3 (3)	0 (0)	0 (0)	
**(*E*,*E*)-α-Farnesene**	<0.5 (<0.5)	7 (4)	1 (1)	1 (1)		1 (<0.5)	15 (12)	3 (3)	2 (1)	
**Germacrene-D**	1 (1)	0 (0)	0 (0)	0 (0)		1 (<0.5)	0 (0)	0 (0)	0 (0)	
**unknown nST 8a**	<0.5 (<0.5)	0 (0)	0 (0)	0 (0)		<0.5 (<0.5)	0 (0)	0 (0)	0 (0)	
**unknown nST 9**	<0.5 (<0.5)	0 (0)	0 (0)	0 (0)		<0.5 (<0.5)	0 (0)	0 (0)	0 (0)	
**unknown nST 10**	<0.5 (<0.5)	0 (0)	0 (0)	0 (0)		<0.5 (<0.5)	0 (0)	0 (0)	0 (0)	
**γ-Cadinene**	<0.5 (<0.5)	<0.5 (<0.5)	<0.5 (<0.5)	<0.5 (<0.5)		<0.5 (<0.5)	<0.5 (<0.5)	1 (1)	<0.5 (<0.5)	
**δ-Cadinene**	1 (<0.5)	1 (<0.5)	<0.5 (<0.5)	<0.5 (<0.5)		1 (<0.5)	1 (1)	1 (1)	<0.5 (<0.5)	
**Bisabolene**	0 (0)	<0.5 (<0.5)	0 (0)	<0.5 (<0.5)		0 (0)	1 (1)	0 (0)	1 (<0.5)	

Supplementary Table 9. Actual (non-standardized) and standardized (+30 °C, isoprene also to PAR-level 1000 µmol m^{-2} s^{-1}) emission rates (ng/g-1 DW h-1) of Norway spruce seedlings exposed to elevated ozone and elevated temperature alone or in combination on week 31 in 2010. Compounds are listed in order of their retention time. Isoprene and non-oxygenated monoterpenes in normal font, *oxygenated monoterpenes* in *cursive*, **non-oxygenated sesquiterpenes (nST) emboldened**, ***GLVs and methylsalicylate emboldened, cursive***. AOAT = ambient ozone, ambient temperature, AOET = ambient ozone, elevate temperature, EOAT = elevated ozone, ambient temperature, EOET = elevated ozone, elevated temperature. Values are means (SE) of four treatment replicates. P-values < 0.05 for the main effects of warming (T) and ozone (O) and P-values <0.1 for the TxO interaction from Mixed Models ANOVA are shown. Significant (*P*<0.05, Bonferroni test) simple main effects of interactions explained in parentheses (e.g., T↓+O = warming reduces under elevated ozone, O↑-T = elevated ozone increases in ambient temperature). Increasing ↑ and decreasing ↓ main effects shown when interaction not found. Tr: *P*-values from Kruskal-Wallis test (differences between treatments, when *P*<0.05 for Bonferroni test).

	Non-standardized					Standardized				
Compound	AOAT	AOET	EOAT	EOET	*P*	AOAT	AOET	EOAT	EOET	*P*
Isoprene	70 (44)	84 (76)	0 (0)	108 (63)		321 (249)	64 (58)	0 (0)	64 (37)	TxO:0.099 (O↓ -T)
Tricyclene	6 (1)	6 (1)	3 (1)	5 (1)		9 (2)	3 (1)	2 (1)	3 (1)	T:0.027, O: 0.020, TxO: 0.020 (O↓-T, T↓ -O)
α-Pinene	131 (51)	166 (43)	58 (12)	265 (93)	T: 0.039↑	184 (82)	81 (22)	45 (13)	146 (59)	TxO:0.070 (O↓ -T)
Camphene	41 (10)	33 (7)	27 (6)	44 (7)		63 (18)	17 (4)	21 (5)	24 (5)	T:0.047, TxO: 0.015 (T↓ -O, O↓ -T)
Sabinene	9 (5)	18 (11)	11 (6)	12 (4)		10 (4)	7 (3)	9 (5)	6 (2)	
β-Pinene	120 (65)	159 (52)	56 (14)	279 (138)		139 (59)	72 (19)	41 (12)	157 (100)	
Myrcene	66 (51)	123 (57)	70 (20)	164 (51)		65 (40)	50 (16)	51 (17)	92 (38)	
(Z)-Hexenyl-acetate	<0.5 (<0.5)	4 (4)	1 (1)	6 (5)		1 (1)	2 (2)	1 (1)	2 (2)	
α-Phellandrene	2 (1)	4 (2)	3 (1)	4 (1)		3 (1)	1 (1)	2 (1)	2 (1)	
α-Terpinene	1 (1)	2 (1)	2 (1)	2 (<0.5)		1 (<0.5)	1 (<0.5)	2 (1)	1 (<0.5)	
3-Carene	182 (119)	273 (192)	283 (209)	82 (36)		197 (96)	92 (56)	246 (202)	46 (23)	
Cymene	2 (1)	4 (1)	4 (2)	5 (1)		2 (1)	2 (<0.5)	3 (1)	2 (1)	
Limonene	176 (91)	812 (634)	367 (156)	556 (245)		198 (76)	148 (50)	300 (159)	287 (132)	
β-Phellandrene	29 (21)	66 (28)	33 (10)	61 (27)		35 (17)	27 (7)	24 (9)	27 (12)	
1,8-Cineole	16 (7)	33 (10)	12 (3)	26 (12)		23 (12)	15 (6)	9 (3)	11 (4)	
γ-Terpinene	2 (2)	6 (3)	5 (3)	3 (1)		2 (1)	2 (1)	4 (3)	2 (<0.5)	
Terpinolene	13 (10)	30 (22)	36 (23)	20 (6)		13 (8)	10 (6)	32 (22)	9 (1)	
Linalool	0 (0)	8 (4)	10 (10)	19 (12)	T:0.020 ↑	0 (0)	3 (1)	8 (8)	7 (3)	
Camphor	2 (2)	25 (15)	7 (2)	13 (6)	T:0.043 ↑	2 (2)	10 (5)	6 (2)	6 (3)	
Borneol	1 (1)	3 (2)	1 (1)	4 (2)		1 (1)	1 (1)	1 (1)	2 (1)	
Pinocarvone	0 (0)	0 (0)	0 (0)	0 (0)		0 (0)	0 (0)	0 (0)	0 (0)	

Supplementary Table 9 continues

	Non-standardized					Standardized				
Compound	AOAT	AOET	EOAT	EOET	*P*	AOAT	AOET	EOAT	EOET	*P*
Isopinocamphone	0 (0)	0 (0)	0 (0)	0 (0)		0 (0)	0 (0)	0 (0)	0 (0)	
Terpinen-4-ol	0 (0)	1 (1)	0 (0)	2 (2)		0 (0)	<0.5 (<0.5)	0 (0)	1 (1)	
α-Terpineol	0 (0)	2 (1)	1 (1)	7 (5)		0 (0)	1 (<0.5)	1 (1)	2 (2)	
Methylsalicylate	0 (0)	13 (10)	0 (0)	21 (9)	T:0.014 ↑	0 (0)	4 (2)	2 (2)	8 (3)	T: 0.023↑
Myrtenal	0 (0)	0 (0)	0 (0)	0 (0)		0 (0)	0 (0)	0 (0)	0 (0)	
Berbenone	0 (0)	0 (0)	0 (0)	0 (0)		0 (0)	0 (0)	0 (0)	0 (0)	
Piperitone	0 (0)	0 (0)	0 (0)	0 (0)		0 (0)	0 (0)	0 (0)	0 (0)	
Bornylacetate	17 (7)	12 (4)	14 (5)	18 (5)		22 (7)	6 (1)	12 (5)	10 (3)	
**α-Cubebene**	1 (1)	0 (0)	0 (0)	<0.5 (<0.5)		1 (1)	0 (0)	0 (0)	<0.5 (<0.5)	
**Longipinene**	19 (17)	9 (6)	8 (6)	6 (3)		15 (11)	2 (1)	7 (6)	2 (1)	
**α-Ylangene**	3 (3)	2 (1)	2 (2)	3 (1)		3 (2)	1 (<0.5)	2 (2)	1 (1)	
**Copaene**	0 (0)	<0.5 (<0.5)	0 (0)	0 (0)		0 (0)	<0.5 (<0.5)	0 (0)	0 (0)	
**Longicyclene**	4 (4)	1 (1)	2 (1)	1 (1)		3 (2)	<0.5 (<0.5)	1 (1)	<0.5 (<0.5)	
**β-Bourbobene**	4 (4)	2 (1)	0 (0)	3 (1)	TxO:0.055 (T↑+O)	3 (2)	1 (<0.5)	0 (0)	1 (<0.5)	TxO:0.033
**unknown nST 1**	1 (1)	3 (3)	0 (0)	0 (0)		1 (1)	<0.5 (<0.5)	0 (0)	0 (0)	
**unknown nST 2**	1 (1)	3 (2)	<0.5 (<0.5)	1 (1)		1 (1)	1 (1)	<0.5 (<0.5)	<0.5 (<0.5)	
**Isoledene**	0 (0)	0 (0)	1 (1)	1 (1)		0 (0)	0 (0)	1 (1)	<0.5 (<0.5)	
**unknown nST 3**	1 (1)	<0.5 (<0.5)	0 (0)	0 (0)		<0.5 (<0.5)	<0.5 (<0.5)	0 (0)	0 (0)	
**unknown nST 4**	0 (0)	0 (0)	0 (0)	<0.5 (<0.5)		0 (0)	0 (0)	0 (0)	<0.5 (<0.5)	
**Longifolene**	14 (11)	12 (10)	8 (5)	8 (4)		12 (8)	2 (1)	6 (5)	2 (1)	
**unknown nST 5**	0 (0)	3 (3)	0 (0)	0 (0)		0 (0)	<0.5 (<0.5)	0 (0)	0 (0)	
**β-Caryophyllene**	4 (2)	13 (9)	1 (1)	3 (2)		3 (1)	1 (1)	<0.5 (<0.5)	1 (<0.5)	
**(*E*)-β-farnesene**	3 (3)	6 (3)	9 (6)	33 (17)	T:0.056 ↑	2 (2)	1 (<0.5)	6 (4)	7 (2)	
**β-Cubebene**	0 (0)	0 (0)	0 (0)	1 (1)		0 (0)	0 (0)	0 (0)	<0.5 (<0.5)	
**unknown nST 6**	0 (0)	1 (1)	0 (0)	1 (1)		0 (0)	<0.5 (<0.5)	0 (0)	<0.5 (<0.5)	
**unknown nST 7**	0 (0)	3 (2)	0 (0)	1 (1)		0 (0)	<0.5 (<0.5)	0 (0)	<0.5 (<0.5)	
**α-Humulene**	1 (1)	3 (2)	<0.5 (<0.5)	<0.5 (<0.5)		1 (1)	<0.5 (<0.5)	<0.5 (<0.5)	<0.5 (<0.5)	
**unknown nST 8**	0 (0)	0 (0)	0 (0)	0 (0)		0 (0)	0 (0)	0 (0)	0 (0)	
**(*E*,*E*)-α-Farnesene**	7 (7)	20 (14)	8 (4)	14 (8)		3 (3)	2 (1)	4 (2)	3 (2)	
**Germacrene-D**	0 (0)	<0.5 (<0.5)	0 (0)	0 (0)		0 (0)	<0.5 (<0.5)	0 (0)	0 (0)	
**unknown nST 8a**	0 (0)	<0.5 (<0.5)	0 (0)	0 (0)		0 (0)	<0.5 (<0.5)	0 (0)	0 (0)	
**unknown nST 9**	0 (0)	0 (0)	0 (0)	0 (0)		0 (0)	0 (0)	0 (0)	0 (0)	
**unknown nST 10**	<0.5 (<0.5)	1 (1)	0 (0)	0 (0)		<0.5 (<0.5)	<0.5 (<0.5)	0 (0)	0 (0)	
**γ-Cadinene**	0 (0)	0 (0)	2 (2)	0 (0)		0 (0)	0 (0)	1 (1)	0 (0)	
**δ-Cadinene**	<0.5 (<0.5)	1 (1)	1 (1)	<0.5 (<0.5)		<0.5 (<0.5)	<0.5 (<0.5)	1 (1)	<0.5 (<0.5)	
**Bisabolene**	0 (0)	0 (0)	1 (1)	4 (2)		0 (0)	0 (0)	<0.5 (<0.5)	1 (<0.5)	

Supplementary Table 10. Actual (non-standardized) and standardized (+30 °C, isoprene also to PAR-level 1000 µmol m^{-2} s^{-1}) emission rates (ng/g-1 DW h-1) of Norway spruce seedlings exposed to elevated ozone and elevated temperature alone or in combination on week 36 in 2010. Compounds are listed in order of their retention time. Isoprene and non-oxygenated monoterpenes in normal font, *oxygenated monoterpenes* in *cursive*, **non-oxygenated sesquiterpenes (nST) emboldened**, ***GLVs and methylsalicylate emboldened, cursive***. AOAT = ambient ozone, ambient temperature, AOET = ambient ozone, elevate temperature, EOAT = elevated ozone, ambient temperature, EOET = elevated ozone, elevated temperature. Values are means (SE) of four treatment replicates. P-values < 0.05 for the main effects of warming (T) and ozone (O) and P-values <0.1 for the TxO interaction from Mixed Models ANOVA are shown. Significant (*P*<0.05, Bonferroni test) simple main effects of interactions explained in parentheses (e.g., T↓+O = warming reduces under elevated ozone, O↑-T = elevated ozone increases in ambient temperature). Increasing ↑ and decreasing ↓ main effects shown when interaction not found. Tr: *P*-values from Kruskal-Wallis test (differences between treatments, when *P*<0.05 for Bonferroni test).

	Non-standardized					Standardized				
Compound	AOAT	AOET	EOAT	EOET	*P*	AOAT	AOET	EOAT	EOET	*P*
Isoprene	0 (0)	8 (8)	12 (7)	79 (37)	T:0.088 ↑	0 (0)	43 (43)	95 (69)	680 (302)	
Tricyclene	6 (1)	6 (1)	6 (2)	7 (3)		21 (3)	15 (2)	21 (5)	22 (10)	
α-Pinene	56 (30)	63 (23)	70 (48)	104 (70)		197 (94)	156 (41)	252 (168)	276 (173)	
Camphene	34 (3)	34 84)	40 (10)	56 (24)		127 (5)	87 (13)	145 (37)	165 (77)	
Sabinene*	-	-	-	-		-	-	-	-	
β-Pinene	17 (13)	30 (20)	12 (7)	47 (44)		57 (43)	70 (41)	43 (25)	119 (110)	
Myrcene	5 (1)	14 (8)	7 (3)	13 (6)		18 (3)	33 (15)	25 (11)	37 (17)	
(Z)-Hexenyl-acetate	0 (0)	0 (0)	0 (0)	0 (0)		0 (0)	0 (0)	0 (0)	0 (0)	
α-Phellandrene	<0.5 (<0.5)	3 (2)	2 (1)	2 (2)		1 (1)	7 (4)	7 (5)	4 (4)	
α-Terpinene	1 (<0.5)	5 (3)	2 (2)	1 (1)		2 (1)	12 (7)	7 (5)	3 (3)	
3-Carene	3 (2)	93 (75)	13 (11)	6 (3)		12 (7)	210 (158)	44 (40)	18 (9)	
Cymene	<0.5 (<0.5)	<0.5 (<0.5)	<0.5 (<0.5)	1 (1)		1 (1)	1 (1)	<0.5 (<0.5)	1 (1)	
Limonene	37 (10)	52 (13)	47 (30)	60 (20)		134 (29)	137 (44)	170 (105)	174 (61)	
β-Phellandrene	6 (3)	20 (15)	5 (3)	20 (16)		19 (10)	44 (31)	18 (11)	51 (40)	
1,8-Cineole	1 (<0.5)	3 (1)	4 (2)	4 (3)		5 (1)	9 (2)	15 (7)	12 (9)	
γ-Terpinene	<0.5 (<0.5)	4 (3)	1 (1)	1 (1)		1 (<0.5)	8 (6)	2 (2)	2 (1)	
Terpinolene	<0.5 (<0.5)	7 (6)	4 (3)	5 (3)		1 (<0.5)	16 (14)	9 (7)	9 (7)	
Linalool	0 (0)	0 (0)	0 (0)	0 (0)		0 (0)	0 (0)	0 (0)	0 (0)	
Camphor	0 (0)	0 (0)	1 (<0.5)	<0.5 (<0.5)		0 (0)	0 (0)	2 (1)	<0.5 (<0.5)	
Borneol	0 (0)	0 (0)	0 (0)	0 (0)		0 (0)	0 (0)	0 (0)	0 (0)	
Pinocarvone	0 (0)	0 (0)	0 (0)	0 (0)		0 (0)	0 (0)	0 (0)	0 (0)	
Isopinocamphone	0 (0)	0 (0)	0 (0)	0 (0)		0 (0)	0 (0)	0 (0)	0 (0)	
Terpinen-4-ol	0 (0)	0 (0)	0 (0)	0 (0)		0 (0)	0 (0)	0 (0)	0 (0)	
α-Terpineol	0 (0)	0 (0)	1 (1)	2 (2)		0 (0)	0 (0)	5 (5)	4 (4)	
Methylsalicylate	0 (0)	0 (0)	0 (0)	0 (0)		0 (0)	0 (0)	0 (0)	0 (0)	
Myrtenal	0 (0)	1 (1)	0 (0)	0 (0)		0 (0)	2 (2)	0 (0)	0 (0)	
Berbenone	0 (0)	0 (0)	0 (0)	0 (0)		0 (0)	0 (0)	0 (0)	0 (0)	
Piperitone	0 (0)	0 (0)	0 (0)	0 (0)		0 (0)	0 (0)	0 (0)	0 (0)	
Bornylacetate	5 (1)	6 (1)	3 (1)	11 (5)		17 (3)	15 (<0.5)	9 (3)	34 (17)	

Table 10 continues

	Non-standardized					Standardized				
Compound	AOAT	AOET	EOAT	EOET	*P*	AOAT	AOET	EOAT	EOET	*P*
**α-Cubebene**	0 (0)	0 (0)	0 (0)	0 (0)		0 (0)	0 (0)	0 (0)	0 (0)	
**Longipinene**	0 (0)	0 (0)	<0.5 (<0.5)	1 (1)		0 (0)	0 (0)	2 (2)	4 (4)	
**α-Ylangene**	0 (0)	<0.5 (<0.5)	0 (0)	0 (0)		0 (0)	1 (1)	0 (0)	0 (0)	
**Copaene**	0 (0)	0 (0)	0 (0)	0 (0)		0 (0)	0 (0)	0 (0)	0 (0)	
**Longicyclene**	0 (0)	0 (0)	0 (0)	0 (0)		0 (0)	0 (0)	0 (0)	0 (0)	
**β-Bourbobene**	0 (0)	0 (0)	0 (0)	0 (0)		0 (0)	0 (0)	0 (0)	0 (0)	
**unknown nST 1**	0 (0)	0 (0)	0 (0)	0 (0)		0 (0)	0 (0)	0 (0)	0 (0)	
**unknown nST 2**	0 (0)	0 (0)	0 (0)	0 (0)		0 (0)	0 (0)	0 (0)	0 (0)	
**Isoledene**	0 (0)	1 (1)	0 (0)	0 (0)		0 (0)	2 (2)	0 (0)	0 (0)	
**unknown nST 3**	0 (0)	0 (0)	0 (0)	0 (0)		0 (0)	0 (0)	0 (0)	0 (0)	
**unknown nST 4**	0 (0)	0 (0)	0 (0)	0 (0)		0 (0)	0 (0)	0 (0)	0 (0)	
**Longifolene**	<0.5 (<0.5)	<0.5 (<0.5)	1 (<0.5)	<0.5 (<0.5)		<0.5 (<0.5)	<0.5 (<0.5)	<0.5 (<0.5)	<0.5 (<0.5)	
**unknown nST 5**	0 (0)	0 (0)	0 (0)	0 (0)		0 (0)	0 (0)	0 (0)	0 (0)	
**β-Caryophyllene**	0 (0)	0 (0)	0 (0)	0 (0)		0 (0)	0 (0)	0 (0)	0 (0)	
**(*E*)-β-farnesene**	0 (0)	0 (0)	0 (0)	0 (0)		0 (0)	0 (0)	0 (0)	0 (0)	
**β-Cubebene**	0 (0)	0 (0)	0 (0)	0 (0)		0 (0)	0 (0)	0 (0)	0 (0)	
**unknown nST 6**	0 (0)	0 (0)	0 (0)	0 (0)		0 (0)	0 (0)	0 (0)	0 (0)	
**unknown nST 7**	0 (0)	0 (0)	0 (0)	0 (0)		0 (0)	0 (0)	0 (0)	0 (0)	
**α-Humulene**	0 (0)	0 (0)	0 (0)	0 (0)		0 (0)	0 (0)	0 (0)	0 (0)	
**unknown nST 8**	0 (0)	0 (0)	0 (0)	0 (0)		0 (0)	0 (0)	0 (0)	0 (0)	
**(*E*,*E*)-α-Farnesene**	0 (0)	0 (0)	0 (0)	0 (0)		0 (0)	0 (0)	0 (0)	0 (0)	
**Germacrene-D**	0 (0)	0 (0)	0 (0)	0 (0)		0 (0)	0 (0)	0 (0)	0 (0)	
**unknown nST 8a**	0 (0)	0 (0)	0 (0)	0 (0)		0 (0)	0 (0)	0 (0)	0 (0)	
**unknown nST 9**	0 (0)	0 (0)	0 (0)	0 (0)		0 (0)	0 (0)	0 (0)	0 (0)	
**unknown nST 10**	0 (0)	0 (0)	0 (0)	0 (0)		0 (0)	0 (0)	0 (0)	0 (0)	
**γ-Cadinene**	0 (0)	0 (0)	0 (0)	0 (0)		0 (0)	0 (0)	0 (0)	0 (0)	
**δ-Cadinene**	0 (0)	<0.5 (<0.5)	0 (0)	0 (0)		0 (0)	<0.5 (<0.5)	0 (0)	0 (0)	
**Bisabolene**	0 (0)	0 (0)	0 (0)	0 (0)		0 (0)	0 (0)	0 (0)	0 (0)	

*not detectable due to GC-MS failure
